# Effects of Induced Pluripotent Stem Cell-Derived Astrocytes on Cisplatin Sensitivity in Pediatric Brain Cancer Cells

**DOI:** 10.3390/cancers17060997

**Published:** 2025-03-16

**Authors:** Sonia Kiran, Yu Xue, Drishty B. Sarker, Qing-Xiang Amy Sang

**Affiliations:** 1 Department of Chemistry and Biochemistry, Florida State University, Tallahassee, FL 32306, USA; skiran@fsu.edu (S.K.); yx21@fsu.edu (Y.X.); ds22@fsu.edu (D.B.S.); 2 Institute of Molecular Biophysics, Florida State University, Tallahassee, FL 32306, USA

**Keywords:** induced pluripotent stem cell, astrocyte, atypical teratoid rhabdoid tumor, diffuse intrinsic pontine glioma, brain tumor microenvironment, cisplatin resistance

## Abstract

**Background:** ATRTs and DIPGs are deadly pediatric brain tumors with poor prognosis. These tumors can develop resistance to chemotherapies, which may be significantly influenced by their microenvironment. Since astrocytes are the most abundant glial cell type in the brain microenvironment and may support tumor growth and chemoresistance, this study investigated the effects of induced pluripotent stem cell-derived astrocytes (iPSC-astrocytes) on cisplatin sensitivity in CHLA-05-ATRT and SF8628 (DIPG) cells. iPSCs provide an unlimited and standardized source of nascent astrocytes, which enables modeling the interaction between childhood brain tumor cells and iPSC-astrocytes within a controlled coculture system. **Methods:** To study the effects on tumor growth, the iPSC-astrocytes were cocultured with tumor cells. Additionally, the tumor cells were exposed to various concentrations of cisplatin to evaluate their chemosensitivity in the presence of astrocytes. **Results:** The paracrine interaction of iPSC-astrocytes with tumor cells upregulated astrocyte activation markers GFAP and STAT3 and promoted tumor cell proliferation. Moreover, the cisplatin treatment significantly decreased the viability of CHLA-05-ATRT and SF8628 cells. However, tumor cells exhibited reduced sensitivity to cisplatin in the coculture with iPSC-astrocytes. During cisplatin treatment, DIPG cells in particular showed upregulation of resistance markers, ERK1, STAT3, and MTDH, which are associated with enhanced proliferation and invasion. They also had increased expression of APEX1, which is involved in the base excision repair pathway following cisplatin-induced DNA damage. **Conclusion:** These findings underscore the significance of the tumor microenvironment in modulating tumor cell survival and chemosensitivity.

## Simple Summary

Atypical teratoid rhabdoid tumors (ATRTs) and diffuse intrinsic pontine gliomas (DIPGs) are lethal pediatric brain tumors that can resist chemotherapy and be influenced by their microenvironment. Astrocytes are the key components of the brain tumor microenvironment and can support tumor growth. We investigated the effects of astrocytes on cisplatin sensitivity in pediatric brain cancer cells. The crosstalk between astrocytes and cancer cells activated astrocytes and promoted cancer cell proliferation. Moreover, the tumor cells expressed elevated levels of drug resistance genes in the presence of astrocytes. In conclusion, astrocytes can significantly improve the growth of these tumor cells and modulate their chemosensitivity, highlighting their role in therapeutic resistance.

## 1. Introduction

Malignant pediatric brain tumors are aggressive neoplasms characterized by rapid invasiveness (grade IV) and poor prognosis. Some of these aggressive tumors develop in children below age 3, such as atypical teratoid rhabdoid tumors (ATRTs) [1], and others are often diagnosed in children under age 10, like diffuse intrinsic pontine glioma (DIPG) [2]. ATRT is a grade IV pediatric tumor with a dismal prognosis and low survival rate [3,4]. It may have a neural progenitor origin [5] and develops primarily due to biallelic inactivation of the SWI/SNF-related, matrix-associated, actin-dependent regulator of chromatin, subfamily B, member 1 (*SMARCB1*) tumor suppressor gene [3]. Similarly, DIPG is a high-grade malignant pediatric brain tumor with an infiltrative nature, originating in the ventral pons of the brainstem [6,7,8]. The majority of DIPGs develop due to the substitution of lysine 27 with methionine in histone H3 (H3K27M), leading to their reclassification as “diffuse midline glioma, H3 K27-altered” in the 2021 WHO Classification of Tumors of the Central Nervous System [7,9]. Unfortunately, ATRTs and DIPGs lack effective therapies; therefore, the mean overall survival of these pediatric patients is <18 months [10,11,12].

Multiple FDA-approved drugs are used to treat ATRTs [13] and DIPGs [8], including cisplatin/cis-diamminedichloroplatinum (II) (CDDP) and methotrexate (MTX). Cisplatin has been an integral component in various chemotherapy protocols used for the clinical management of childhood brain malignancies [14,15]. However, some cancer cells develop resistance against cisplatin using multiple mechanisms, such as inhibiting apoptosis, promoting cell survival, or pumping drugs outside cells [16,17]. Upregulation of markers involved in these pathways is observed, e.g., nuclear factor-κB 1 (NFκB1) and signal transducer and activator of the transcription 3 (STAT3) [16,18]. Additionally, other signaling axes, e.g., signal-regulated kinase (ERK)/mitogen-activated protein kinase (MAPK), Metadherin (MTDH), apurinic/apyrimidinic endonuclease-1 (APEX1), etc., also coordinate with each other to promote cisplatin resistance in different cancers [19,20,21,22,23]. However, the potential implications of these pathways for ATRTs’ and DIPGs’ cisplatin resistance remain to be examined.

The brain tumor microenvironment (TME) comprises stromal cells, immune cells, and glial cells that can mediate therapeutic resistance in aggressive brain tumors [24,25]. Astrocytes are among the most abundant glial cells (around 50%) [26]. Although they are activated to protect and repair tissue during brain injury, in brain tumors, they can promote tumor progression, invasion, and therapeutic resistance [27,28]. Studies have shown that astrocytes within the microenvironment of primary brain tumors promote an anti-inflammatory state and facilitate tumor growth and invasiveness [29,30,31]. The coculture of astrocytes with several cancer cell lines also resulted in the activation of survival genes, contributing to resistance to an array of chemotherapeutic agents [32]. Given the limited understanding of how astrocytes contribute to chemoresistance and tumor progression in ATRTs and DIPGs, we hypothesize that iPSC-derived astrocytes may modulate drug sensitivity in these cancer cells by upregulating key resistance pathways, thereby contributing to their chemoresistance.

Human astrocytes from adult brain tissue are challenging to culture and usually do not proliferate beyond a few passages [33,34], limiting their accessibility for research. Moreover, the phenotypic and functional properties of adult astrocytes are different from those of nascent astrocytes in a developing central nervous system [35]. Therefore, to better model the astrocyte–tumor interaction of these childhood brain cancers originating from progenitor cells [36,37], human induced pluripotent stem cell-derived astrocytes (iPSC-astrocytes) can be particularly useful.

iPSC-astrocytes are more relevant to the microenvironment of pediatric brain cancers as they are nascent yet functional glial cells [38,39]. iPSCs can be expanded indefinitely in vitro to support large batches with consistent properties of astrocytes. In contrast, primary astrocytes often vary between batches and may senesce or lose phenotype over passages [40]. Additionally, unlike commercially sourced astrocytes, which may not accurately reflect patient-specific conditions, iPSC-derived astrocytes can be generated from patient-specific iPSCs, retaining genetic and epigenetic characteristics relevant to the tumor microenvironment. Hence, iPSC-astrocytes are a reliable source for modeling pediatric brain astrocytes [34].

This study specifically investigated the effects of iPSC-astrocytes on cisplatin sensitivity in DIPG and ATRT cells. We examined the proliferative capacity of the tumor cells during their paracrine interaction with iPSC-astrocytes. Moreover, upon cisplatin exposure, the sensitivity of tumor cells cocultured with iPSC-astrocytes was also evaluated. We further investigated the markers associated with cisplatin resistance pathways in tumor cells to uncover the potential influence of astrocytes on their survival and chemoresistance. Hence, studying the key markers of cisplatin resistance pathways in ATRT and DIPG cells and investigating how astrocytes affect their regulation may provide better insights for developing effective therapies against these malignancies.

## 2. Materials and Methods

### 2.1. Human iPSC, Normal Human Astrocyte, and Cancer Cell Culture

Human episomal iPSCs (Epi-iPSCs, Thermo Fisher Scientific, Waltham, MA, USA, Cat# A18945) were employed for astrocyte differentiation. These cells are derived from human cord blood CD34+ cells using a three-episomal plasmid system that expresses reprogramming factors (SOX2, OCT4, KLF4, MYC, NANOG, LIN28, and SV40 T antigen; SOKMNLT). The cells were plated on a Matrigel-coated (Life Sciences, Corning, NY, USA, Cat# 354234) 6-well plate (VWR, Suwanee, GA, USA) at a density of 1 × 10^6^ cells per well. The culture medium consisted of mTeSR plus serum-free medium supplemented with mTeSR plus 5x supplement (STEMCELL Technologies, Cambridge, MA, USA). A 10 µM concentration of ROCK inhibitor Y27632 (Sigma-Aldrich, Saint Louis, MO, USA) was added during the first 24 h to enhance cell survival and proliferation. The cells were replated upon reaching confluency, while the remaining cells were frozen in a freezing solution containing 90% Fetal Bovine Serum (FBS, VWR, Radnor, PA, USA) and 10% Dimethyl sulfoxide (DMSO, VWR, Radnor, PA, USA).

This study used a normal human astrocyte (NHA, Lonza bioscience, Westlake, LA, USA) cell line as a control. The cells were cultured in Astrocyte Growth Medium (AGM™ BulletKit™, CC-3186, Greenwood, SC, USA) which contained 500 mL Astrocyte Basal Medium (ABM™, Greenwood, SC, USA) and growth supplements, including recombinant human epidermal growth factor (rhEGF, Greenwood, SC, USA), 1.25 mL of Insulin, 0.5 mL of Ascorbic Acid, 0.5 mL of GA-1000, 5 mL of L-glutamine, and 15 mL of FBS.

CHLA-05-ATRT cells (ATCC^®^ CRL-3037^TM^, ATCC, Manassas, VA, USA) were cultured in suspension on a non-treated surface using Gibco Dulbecco’s Modified Eagle Medium/Nutrient Mixture F-12 (DMEM/F-12, Thermo Fisher Scientific, Waltham, MA, USA) supplemented with 2% B-27 serum-free supplement (Thermo Fisher Scientific, Waltham, MA, USA), 20 ng/mL of epidermal growth factor (EGF, 78006.1, STEMCELL Technologies, Vancouver, BC, Canada), and 20 ng/mL of fibroblast growth factor (FGF)-2 (78003.1, STEMCELL Technologies, Cambridge, MA, USA) [41,42]. The SF8628 Human DIPG H3.3-K27M cell line (Sigma-Aldrich, Cat# SCC127, Saint Louis, MO, USA) was maintained in DMEM-High Glucose (Sigma-Aldrich, Cat# D6546, Saint Louis, MO, USA) medium supplemented with 10% FBS (EMD Millipore, Burlington, MA, USA, Cat# ES-009-B), 2 mM L-Glutamine (EMD Millipore, Burlington, MA, USA, Cat. No. TMS-002-C), and 1X penicillin–streptomycin solution (EMD Millipore, Burlington, MA, USA, Cat. No. TMS-AB2-C) [41]. All cell lines were cultured at 37 °C and 5% CO_2_ in an incubator.

### 2.2. Differentiation of Human iPSCs into Astrocytes

The human iPSCs were differentiated into astrocytes following the published protocol [43]. The iPSCs were seeded in a low attachment plate containing pre-warmed Dulbecco’s Modified Eagle Medium/Nutrient Mixture F-12 (DMEM/F-12) supplemented with 2% B-27 serum-free supplement (Thermo Fisher Scientific, Waltham, MA, USA). The cells were treated with ROCKi (10 μM, STEMCELL Technologies, Cambridge, MA, USA) during the first 24 h to promote proliferation and embryoid body formation. After 6 days, the cells were exposed to fibroblast growth factor (FGF) 2 (10 ng/mL, STEMCELL Technologies, Cambridge, MA, USA), epidermal growth factor (EGF) (10 ng/mL, STEMCELL Technologies, Cambridge, MA, USA), and heparin (5 ug/mL, Sigma-Aldrich, Saint Louis, MO, USA) to facilitate neural progenitor expansion, with half medium change every second day. On day 13, neural progenitor spheres were dissociated using Accutase (STEMCELL Technologies, Cambridge, MA, USA), and the resulting single cells were plated on a T-75 flask coated with Matrigel^®^ Matrix (Corning^®^, Life Sciences, Corning, NY, USA, Cat# 354234). The cells were cultured for 15 days in DMEM/F-12 medium supplemented with 2% B-27, 0.5 μM retinoic acid, and 2 μg/mL heparin (Thermo Fisher Scientific, Waltham, MA, USA). From day 27 to day 40, astrocyte lineage and maturation were induced using the DMEM/F-12 medium supplemented with 2% B-27 and heparin. The differentiated cells were characterized using astrocyte-specific markers. The morphology and functional markers of derived cells were compared with normal human astrocytes, such as calcium-binding protein B (S100B) and glial fibrillary acidic protein (GFAP). Differentiated astrocytes were expanded for 10 days in the same medium supplemented with 2% B-27 and heparin.

### 2.3. Tumor Cell Coculture with iPSC-Astrocytes and Drug Treatment

Cisplatin, or cis-diamminedichloroplatinum (II) (Cat# 225150, Thermo Fisher Scientific, Waltham, MA, USA), stock solution (500 µM) was prepared by dissolving the powder in phosphate-buffered saline (PBS, pH 7.4) at room temperature (RT). Similarly, methotrexate (AAJ63075MC, Thermo Fisher Scientific, Waltham, MA, USA) stock solution (50 mM) was prepared by dissolving the powder in 0.1 M sodium hydroxide (NaOH) at RT. ATRT and glioma cells were exposed to multiple concentrations of these drugs—0.5 μM, 1 μM, 25 μM, 50 μM, and 100 μM—for 48 h. To measure IC50 using dose–response analysis, these cancer cells were exposed to 0.001, 0.01, 0.1, 1, 10, and 100 μM of CDDP and 0.005, 0.05, 0.5, 5, 50, and 500 μM of MTX. The negative and vehicle controls for the drug-treated experiments were tumor cells cultured in PBS-added media (control for CDDP), NaOH-added media (control for MTX), and media only for 48 h.

To establish the coculture system, tissue culture plate inserts with polyester membrane (VWR, Radnor, PA, USA,) were utilized. iPSC-astrocytes were plated on these inserts, and tumor cells were seeded onto the lower chamber at an astrocyte-to-tumor cell ratio of 1:2. The coculture was incubated for 48 h to evaluate the cytotoxic effects of the drugs. The common negative control for all the coculture systems was tumor cells without astrocytes. Additionally, the controls for the drug-treated cocultures were the cocultures without drugs, without astrocytes, and with vehicle controls of each drug.

### 2.4. 3-(4,5-Dimethylthiazol-2-yl)-2,5-diphenyltetrazolium Bromide (MTT) Assay

The growth effect of iPSC-astrocytes on cancer cells and the effect of drugs on the viability of astrocyte-cocultured and monoculture ATRT and glioma cells were tested using an MTT assay. Pediatric tumor cells were seeded in triplicate for each experimental condition in surface-treated 96-well plates. After 8 h, these cells were exposed to various concentrations of drugs for 48 h. The cells were washed twice with PBS after drug treatment. MTT (Cat# M6494, Thermo Fisher Scientific, Waltham, MA, USA) stock solution (5 mg/mL) was prepared in PBS and diluted 1:10 with cell culture media. The washed cells were then incubated with the diluted MTT solution for 40–45 min, allowing the formation of purple formazan crystals as an indicator of metabolic activity. DMSO was added to each well to solubilize the crystals. Absorbance was measured at 570 nm using a spectrophotometer and normalized with the number of cells seeded for the experiment to find viable and proliferating cells. Vehicle (for CDDP, the PBS in media, and for MTX, 0.1 M NaOH in media)-treated tumor cells were used as controls. The relative percentage of cell viability for each condition was calculated using the following formula:

$$\mathrm{Relative}\;\mathrm{percentage}\;\mathrm{cell}\;\mathrm{viability}(Y)=\left(\frac{\left(\mathrm{viability}\;\mathrm{of}\;\mathrm{cells}\;\mathrm{exposed}\;\mathrm{to}\;\mathrm{drugs}\right)}{\mathrm{viability}\;\mathrm{of}\;\mathrm{unexposed}\;\mathrm{cells}}\right)\times 100$$

### 2.5. Immunocytochemistry (ICC)

The tumor cells were seeded at a density of 7000 cells per well on a Matrigel-coated 96-well plate and maintained in their respective media for 24 h. The cells were then fixed with 4% paraformaldehyde for 30 min at room temperature (RT), followed by two PBS washes. The cells were permeabilized with 0.5% Triton X-100 for 10 min at RT for the intracellular markers. After permeabilization, two additional PBS washes were performed, followed by blocking the non-specific binding sites for 45 min with a blocking buffer containing 2% FBS in PBS. Primary antibodies (Supplementary Table S1) associated with each marker were used to bind with cells overnight at 4 °C. Secondary staining was performed using Alexa Fluor 488 goat anti-mouse IgG, IgM(H+L) (Cat#A-10680, Thermo Fisher Scientific, Waltham, MA, USA) or Alexa Fluor 594 goat anti-rabbit IgG(H+L) (Cat#A-11012, Thermo Fisher Scientific, Waltham, MA, USA) antibodies for 40 min at RT. After the secondary staining, nuclei were counterstained with Hoechst 33342 (blue, Cat# 62249, Thermo Fisher Scientific, Waltham, MA, USA). A fluorescent microscope (Keyence, BZ-X800 microscope, Keyence Corporation of America, Itasca, IL, USA) used to capture immunofluorescence images, which were analyzed using the ImageJ software (version 1.54p, NIH and LOCI, University of Wisconsin, USA, https://imagej.net/ij/ accessed on: 23 August 2024).

### 2.6. Reverse Transcription-Polymerase Chain Reaction (RT-PCR)

The total mRNA was isolated using the RNeasy^®^ Mini Kit (Qiagen, Valencia, CA, USA) and further concentrated and purified with the DNA-free RNA Kit (Zymo, Irvine, CA, USA). For the reversed transcription, approximately 1 μg of total RNA was anchored on oligo-dT primers and reverse-transcribed to complementary DNA (cDNA) using Superscript™ III Reverse Transcriptase (Invitrogen, Carlsbad, CA, USA). The primers specific to target cDNAs (Supplementary Table S2) were designed using the Primer-BLAST tool (https://www.ncbi.nlm.nih.gov/tools/primer-blast/ accessed on: 23 August 2024 ), and their melting points were checked using NetPrimer (https://www.premierbiosoft.com/netprimer/ accessed on: 23 August 2024). β-actin gene was used as the endogenous control. Quantitative ABI7500 instrument (Applied Biosystems, Foster City, CA, USA) and SYBR Green PCR Master Mix (QuantaBio, Beverly, MA, USA) were used to perform the RT-PCR experiment. The thermal cycling conditions were as follows: 95 °C for 10 min, followed by 40 amplification cycles of 95 °C for 15 s, 55 °C for 30 s, and 68 °C for 30 s. The target genes’ cyclic threshold (Ct) values were normalized to the C_t_ values of β-actin to find ΔC_t_. The ΔC_t_ values of genes in all the drug-treated samples were compared to the untreated sample to measure the fold change in gene expression using the following formula, 2^−(ΔCt sample − ΔCt control)^.

### 2.7. Quantitative Analysis of Marker Expression Using Flow Cytometry

To prepare the flow cytometry samples, the cells were detached using Accutase and fixed with 10% neutral buffered formalin (VWR) for 30 min at RT. Following fixation, the cells investigated for the intracellular markers were permeabilized with 100% cold methanol for 10 min. After permeabilization, the cells were washed twice with PBS and were treated for 40 min with a blocking buffer containing 2% FBS in PBS to block non-specific binding sites. All the samples were incubated with primary antibodies of interest overnight at 4 °C. The Next day, the primary antibody was removed, and the cells were given 2–3 PBS washes. Secondary antibodies—Alexa Fluor 488 goat anti-mouse IgG, IgM(H+L) (Cat#A-10680, Thermo Fisher Scientific, Waltham, MA, USA) or Alexa Fluor 594 goat anti-rabbit IgG (H+L) (Cat#A-11012, Thermo Fisher Scientific, Waltham, MA, USA)—were added, and the samples were incubated for 40 min at RT. After another 2–3 PBS washes, the samples were analyzed using a BD FACSCanto™ II flow cytometer (Becton Dickinson, Tustin, CA, USA) and Cytek Aurora™ (Cytek Biosciences, Bethesda, MD, USA). The results were analyzed against isotype controls using the FlowJo software (Version 10.8.1, Ashland, OR USA).

### 2.8. Statistical Analysis

Each experiment was carried out in triplicate, and the results were presented as [mean ± standard deviation]. Statistical analysis was conducted using one-way analysis of variance (ANOVA) followed by Fisher’s least significant difference (LSD) post hoc tests to compare group means. A *p*-value of <0.05 was considered statistically significant.

## 3. Results

### 3.1. Effects of Crosstalk Between iPSC-Astrocytes and Brain Tumor Cells

#### 3.1.1. Characterization of iPSC-Astrocytes and Comparison of Their Phenotype with NHA

**Figure 1:**
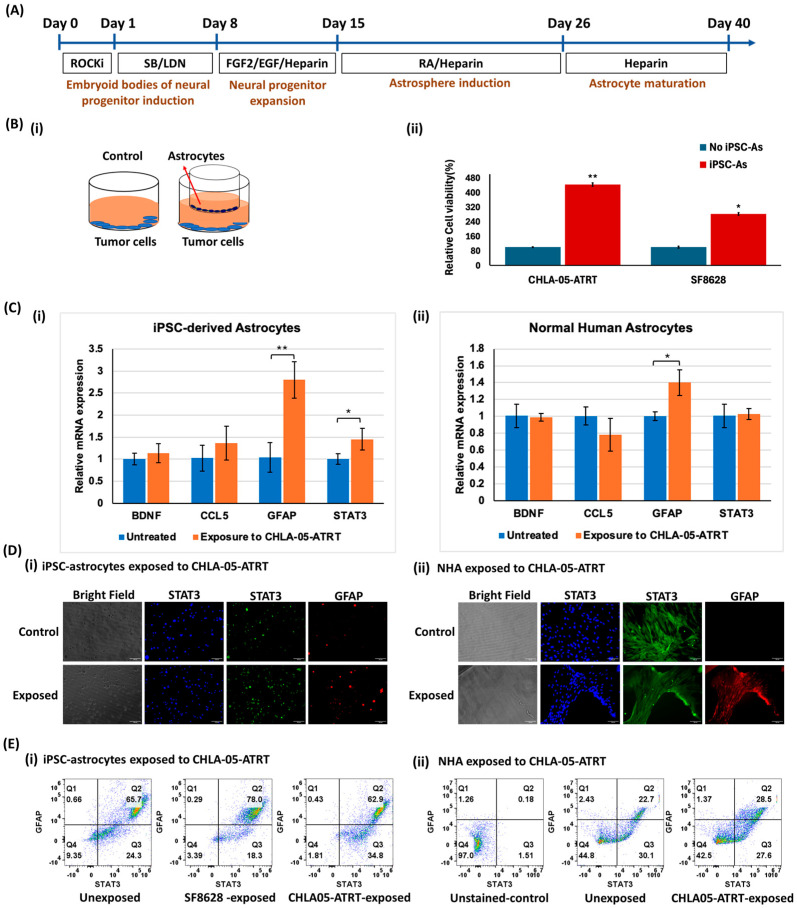
(**A**) Astrocyte differentiation from induced pluripotent stem cells (iPSCs). The schematic diagram shows the derivation of astrocytes from iPSCs. Rocki: rho-associated protein kinase inhibitor Y27632; FGF: fibroblast growth factor; EGF: epidermal growth factor; RA: retinoic acid. (**B**) Effect of iPSC-astrocytes on the proliferation of pediatric brain tumor cells. (**i**) The diagram represents the coculture system for the paracrine interaction of iPSC-astrocytes and tumor cells. (**ii**) The proliferation rates of DIPG and CHLA-05-ATRT cells increased significantly after coculture with astrocytes. (**C**) Effects of cancer cell exposure on (**i**) iPSC-astrocytes and (**ii**) normal human astrocytes (NHA). The relative mRNA expression of markers associated with the pro-inflammatory state of both iPSC-astrocytes and normal human astrocytes was measured after their exposure to tumor cells. The protein expression levels of STAT3 and GFAP shown in (**D**) fluorescent images and (**E**) flow cytometry data indicate higher percent expression in (**i**) iPSC-astrocytes and (**ii**) NHA upon coculture with cancer cells. GFAP: Glial fibrillary acidic protein, CCL5: c-c chemokine ligand 5, BDNF: Brain-derived neurotrophic factor. The Scale bar in (**D**) is 100 μm. The error bars represent the standard deviation in triplicate readings, and statistical significance is shown by * and ** for *p*-values less than 0.05 and 0.01, respectively.

The human iPSCs were differentiated into astrocytes following the differentiation protocol in Figure 1A. The iPSCs initially progressed through the neural progenitor stage, followed by the formation of astrocyte precursors, and ultimately matured into fully differentiated astrocytes over 40 days. The changing morphology of iPSCs at various stages was monitored using phase-contrast imaging (Supplementary Figures S1A(i) and S2A). To initiate the differentiation, iPSCs were cultured in the low attachment plate to form embryoid bodies committed to neural progenitor cells. Upon lineage specification of these progenitors towards astrocytes, the cells exhibited the morphology of normal human astrocytes (Supplementary Figure S1C(i)). The differentiated astrocytes were characterized by the expression of markers (Supplementary Table S3), including chondroitin sulfate proteoglycan (CSPG), glial fibrillary acidic protein (GFAP), and S100 calcium-binding protein B (S100B). The derived cells highly expressed astrocyte-specific markers with 78% expression of S100B and 59% expression of GFAP (Supplementary Figures S1 and S2). For comparison, the morphology and phenotypic properties of normal human astrocytes were also investigated (Supplementary Figures S1C and S3). The differentiated cells were further expanded for 10 days before their use in coculture experiments.

#### 3.1.2. Tumor Cell and iPSC-Astrocyte Paracrine Interaction Resulted in Astrocyte Activation and Tumor Cell Proliferation

A cell viability assay was performed to investigate the influence of iPSC-derived astrocytes on tumor cell proliferation. The iPSC-astrocytes were cocultured at a ratio of 1:2 (astrocyte/tumor cells) with SF8628 cells and CHLA-05-ATRT cells separately for 48 h to assess the effect of paracrine interactions on tumor cell growth (Figure 1B(i)). The results showed a significant increase in the proliferation of tumor cells when exposed to iPSC-astrocytes. CHLA-05-ATRT cells showed a 4.43-fold higher proliferation rate, while DIPG cells exhibited a 2.8-fold increase (Figure 1B(ii)).

To study the molecular effects of tumor cells on human iPSC-derived astrocytes, tumor cells were plated on inserts, and astrocytes were seeded in the lower chamber at a 2:1 ratio (astrocyte/tumor cells). After 48 h of exposure to tumor cells, iPSC-derived astrocytes displayed the upregulation of pro-inflammatory markers, GFAP and STAT3, by 2.8-fold and 2-fold, respectively (Figure 1C(i)), suggesting their activation [29]. The normal human astrocyte cell line, studied as a control, also showed significant upregulation of GFAP upon coculture with tumor cells, indicating their pro-inflammatory state (Figure 1C(ii)). The protein expression levels of these markers also increased in both iPSC-astrocytes and NHA upon coculture with cancer cells (Figure 1D,E and Supplementary Figures S4 and S5).

### 3.2. Cytotoxic Effect of Anti-Cancer Drugs on Malignant Pediatric Brain Tumor Cells

**Figure 2:**
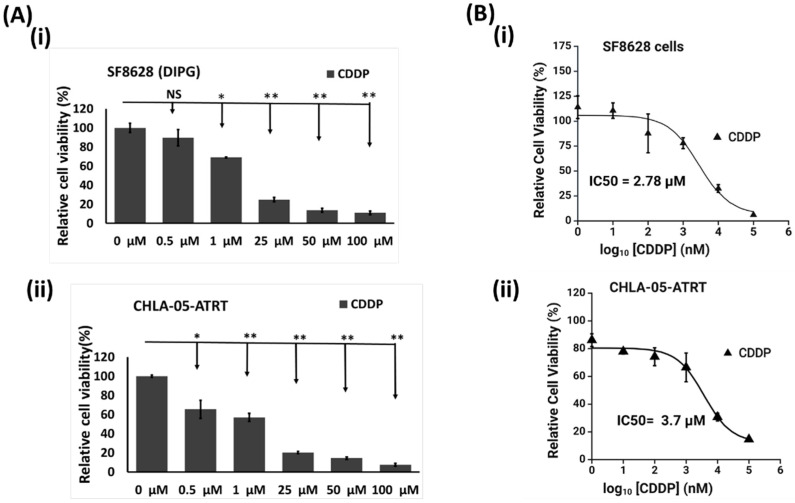
Relative viability study in ATRT and DIPG cells exposed to cisplatin at various concentrations. (**A**) Bar graphs indicate the percent cytotoxic effects of cisplatin (CDDP) on the viability of (**i**) SF8628 and (**ii**) CHLA-05-ATRT cells. (**B**) The dose–response curve of cisplatin indicating its cytotoxicity towards (**i**) SF8628 and (**ii**) CHLA-05-ATRT cells. The error bars represent triplicate readings, and statistical significance is shown by * and ** for *p*-values less than 0.05 and 0.01, respectively. NS: Not Significant. The inhibition dose–response curves are plotted and IC50 was calculated [46] using Biorender.com.

The anti-tumor activity of FDA-approved drugs such as cisplatin and methotrexate has been tested in solid cancers, melanomas, lymphomas, and many other cancers, especially in combination with other therapies [44,45]. We exposed CHLA-05-ATRT and SF8628 cells to multiple concentrations of these drugs to study their effects on these malignant brain tumor cells (Figure 2 and Supplementary Figure S6).

#### 3.2.1. Cisplatin or Cis-Diamminedichloroplatinum (II)

The cytotoxicity of cisplatin was tested in SF8628(DIPG) cells and CHLA-05-ATRT cells. With increased dosage, the drug elicited decreased viability in both cell lines. In SF8628 cells, the viability was around 13% to 10% at 50 and 100 μM (Figure 2A(i),B(i)). Even at 25 μM concentration of cisplatin, the tumor cell survival decreased to 24%. The data suggested a strong anti-tumor activity of cisplatin in SF8628 cells. Similarly, CHLA-05-ATRT cells were also sensitive to cisplatin as they displayed a significant decrease in viability (Figure 2A(ii),B(ii)). At 25 μM concentration of cisplatin, the tumor cell growth decreased to 20% and went down to 14% and 7% at 50 μM and 100 μM, respectively. This decrease in the viability of CHLA-05-ATRT and SF8628 cells indicates the efficacy of cisplatin against these tumor cells (Figure 2).

#### 3.2.2. Methotrexate

Compared to cisplatin, SF8628 and CHLA-05-ATRT cells displayed relatively less sensitivity to methotrexate (Supplementary Figure S6). The IC50 of MTX in both cancer cells was higher than that of cisplatin (Figure 2 and Supplementary Figure S6). Unlike cisplatin, the significant effect of MTX on tumor cell viability was only observed at higher concentrations (Supplementary Figure S6).

### 3.3. Activity of Anti-Tumor Drugs in ATRT and DIPG Cells Cocultured with iPSC-Astrocytes

**Figure 3:**
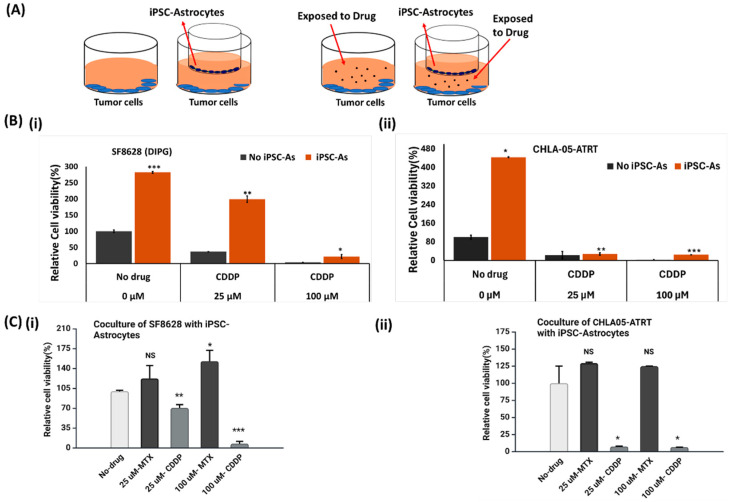
Effect of the crosstalk between iPSC-astrocytes and pediatric brain cancer cells on the cytotoxicity of anti-cancer drugs. (**A**) The diagram of the coculture system represents the paracrine interaction of iPSC-astrocytes and tumor cells during drug exposure. The culture was incubated for 48 h before the tumor cell viability test. (**B**) Upon exposure to cisplatin (CDDP) at 25 μM and 100 μM, the viability of (**i**) DIPG cells and (**ii**) CHLA05-ATRT cells increased significantly in the coculture with astrocytes. (**C**) The effects of both CDDP and MTX at 25 μM and 100 μM on the viability of (**i**) SF8628 and (**ii**) CHLA-05-ATRT cells during their coculture with astrocytes. The error bars represent the standard deviation in triplicate readings, and statistical significance is shown by *, **, and *** for *p*-values less than 0.05, 0.01 and 0.001, respectively. NS: Not Significant.

To investigate the effect of iPSC-astrocytes on the anti-tumor activity of cisplatin and methotrexate, an indirect coculture system was established (tumor cells/astrocytes = 2:1) (Figure 3A). This system was tested under four conditions: tumor cells only, tumor cells cocultured with astrocytes, tumor cells exposed to the drugs, and tumor cells exposed to the drugs within the coculture system (Figure 3A). Cisplatin and methotrexate were added to their respective culture conditions at final concentrations of 25 and 100 μM. The tumor cells were cultured in these conditions for 48 h before measuring their viability. The results indicated a significant increase in tumor cell growth in the presence of iPSC-astrocytes (Figure 3 and Supplementary Figure S7). The tumor cells exposed to drugs only showed a significant reduction in growth, especially the cisplatin-treated cells. However, tumor cells cocultured with astrocytes were less sensitive to these drugs. The results of SF8628 cell viability (Figure 3B(i) and Supplementary Figure S7B) suggest that their interaction with astrocytes developed resistance against drugs, lowering their sensitivity. At 100 µM, cisplatin lowered the percent viability of SF8628 (DIPG) cells to 4–10%. In contrast, in the cisplatin-astrocyte (CDDP-As) condition, the viability was 22% (Figure 3B(i)), implying the pro-tumorigenic effects of astrocytes. Compared to DIPG cells, CHLA-05-ATRT cells were more sensitive to the drugs in the coculture with astrocytes. Although astrocytes increased the viability of CHLA-05-ATRT cells under methotrexate treatment (Figure 3C(ii)), their growth-promoting effects in cisplatin-treated conditions were less pronounced (Figure 3B(ii),C(ii)).

### 3.4. iPSC-Astrocytes Modulated Cisplatin Resistance and Proliferation Pathways in ATRT and DIPG Cells

**Figure 4:**
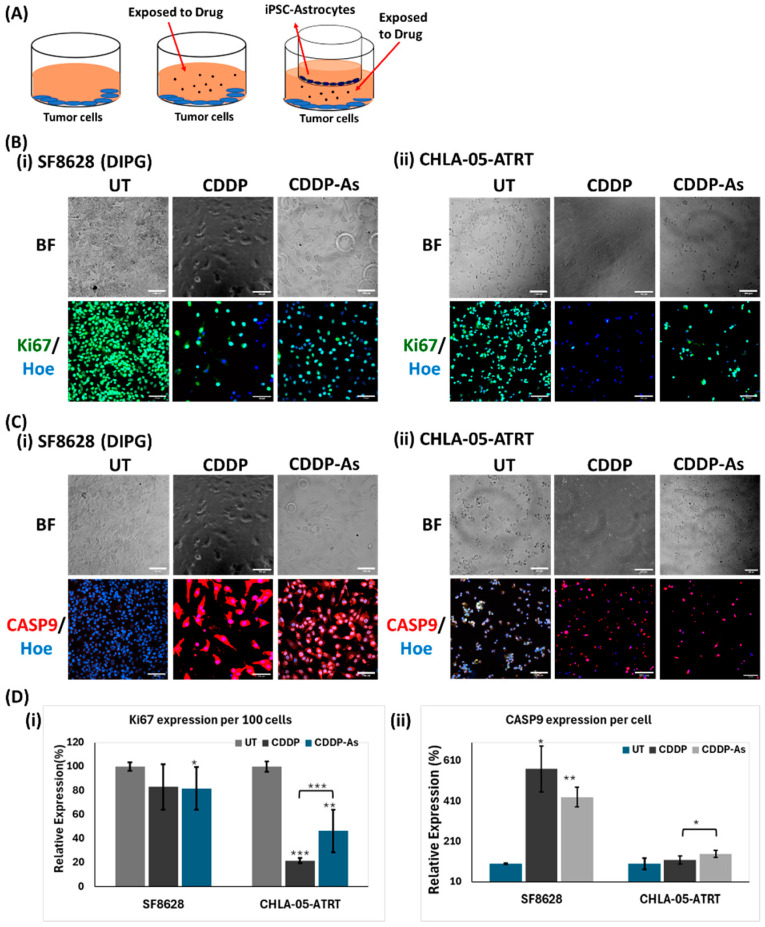
Proliferation and apoptosis markers in pediatric brain tumor cells exposed to cisplatin and iPSC-astrocytes. (**A**) The diagram of the cell culture model was followed to set up all three culture conditions. The expression of (**B**) Ki-67 and (**C**) Caspase9 (Casp9) in (**i**) SF8628 (DIPG) and (**ii**) CHLA-05-ATRT cells exposed to untreated, cisplatin, and cisplatin–astrocyte treated conditions. (**D**) The expression of (**i**) Ki-67 per 100 cells and (**ii**) Casp9 per cell is quantified using ImageJ software. The images were captured with 200× magnification; the scale bar is 100 μm. Hoe: Hoechst 33342; BF: Brightfield; UT: untreated cell; CCDP-As: cells exposed to cisplatin and iPSC-astrocytes. The error bars represent triplicate readings, and statistical significance is shown by *, **, and *** for *p*-values less than 0.05, 0.01, and 0.001, respectively.

Following 48 h of exposure, the tumor cells were analyzed for the expression of apoptosis and proliferation markers, such as Caspase-9 and Ki-67 (Figure 4), as well as markers associated with cisplatin resistance, including APEX1, MTDH, ERK1, and STAT3. Caspase-9 is involved in caspase-dependent apoptosis [47], whereas Ki-67 is a marker of tumor cell proliferation [48,49]. After cisplatin treatment, a reduction in cell density was observed, alongside a decrease in Ki-67 expression per 100 cells (Figure 4B,D(i)). Both cisplatin-exposed (CDDP) and cisplatin–astrocyte-exposed (CDDP-As) SF8628 (DIPG) and CHLA-05-ATRT cells showed higher expression of apoptosis marker Caspase-9 (Figure 4C,D(ii)).

**Figure 5:**
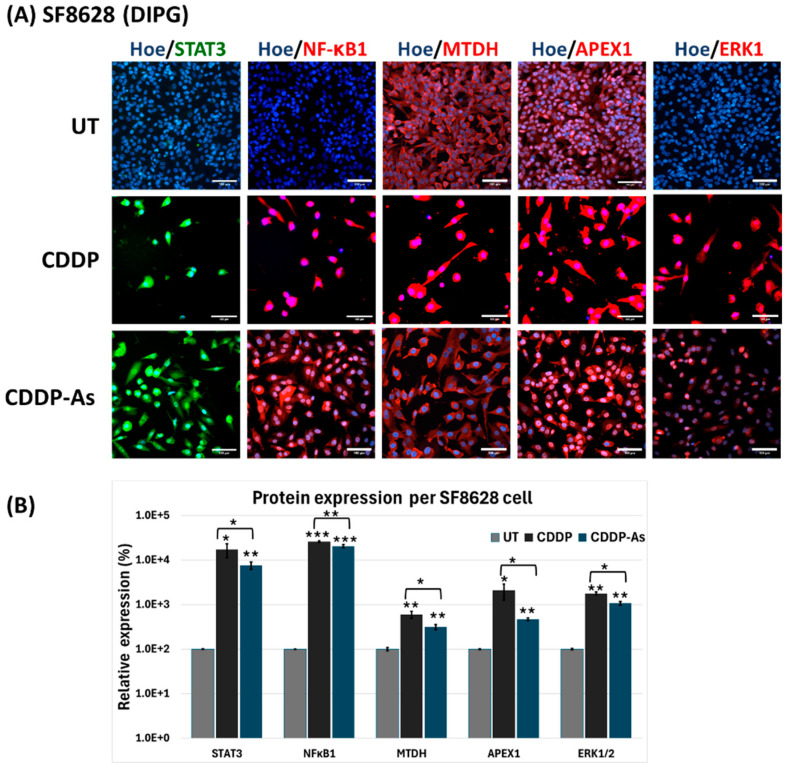
Markers involved in chemoresistance and tumorigenesis after exposure of SF8628 cells to various conditions of cisplatin and iPSC-astrocytes. (**A**) The immunofluorescent images show expression of STAT3, NFκB1, MTDH, APEX1, and ERK1 with the co-expression of Hoechst 33342 in SF8628 cells and (**B**) their quantification by measuring expression of signal intensity per SF8628 cell using ImageJ. The images were captured with 200× magnification, and the scale bar is 100 μm. Hoe: Hoechst 33342, STAT3: Signal transducer and activator of transcription 3, NFκB1: Nuclear factor-κB 1, APEX1: Apurinic/apyrimidinic endonuclease 1, ERK1: Extracellular signal-regulated kinase 1, MTDH: Metadherin. BF: Brightfield; UT: untreated cells; CCDP-As: cells exposed to cisplatin and iPSC-astrocytes. The error bars represent triplicate readings, and statistical significance is shown by *, **, and *** for *p*-values less than 0.05, 0.01, and 0.001, respectively.

**Figure 6:**
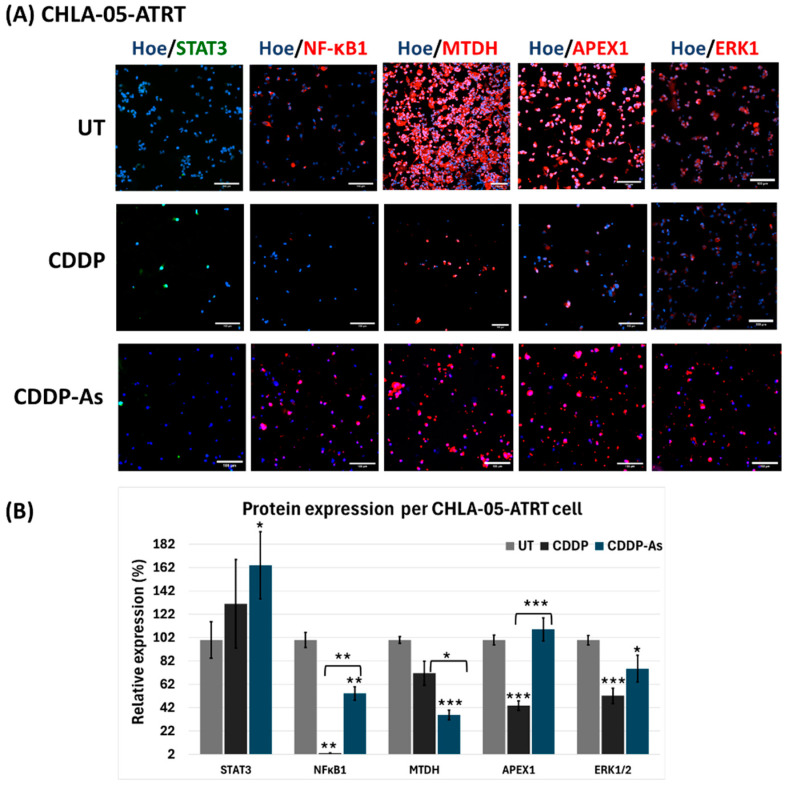
Markers involved in chemoresistance and tumorigenesis after exposure of CHLA-05-ATRT cells to various conditions of cisplatin and iPSC-astrocytes. (**A**) The immunofluorescent images show expression of STAT3, NFκB1, MTDH, APEX1, and ERK1 with the co-expression of Hoechst 33342 in CHLA-05-ATRT cells and (**B**) their quantification by measuring expression of signal intensity per cell using ImageJ. The images were captured with 200× magnification; the scale bar is 100 μm. Hoe: Hoechst 33342, STAT3: Signal transducer and activator of transcription 3, NFκB1: Nuclear factor-κB 1, APEX1: Apurinic/apyrimidinic endonuclease 1, ERK1: Extracellular signal-regulated kinase 1, MTDH: Metadherin. BF: Brightfield; UT: untreated cells; CCDP-As: cells exposed to cisplatin and iPSC-astrocytes. The error bars represent triplicate readings, and statistical significance is shown by *, **, and *** for *p*-values less than 0.05, 0.01, and 0.001, respectively.

**Figure 7:**
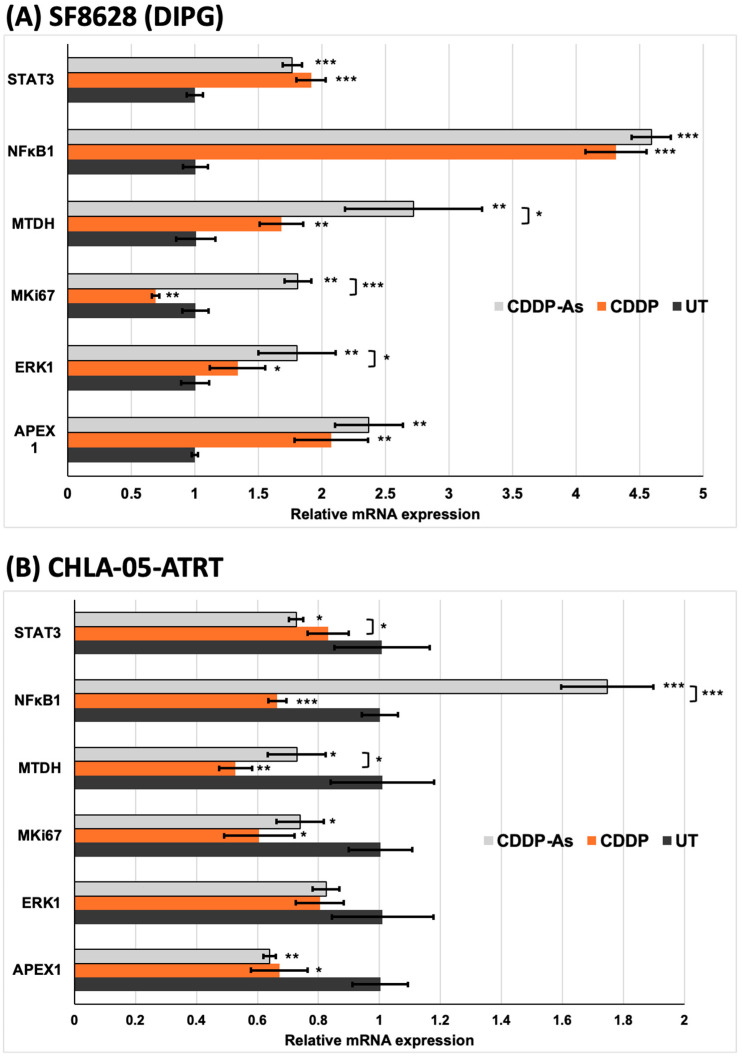
The mRNA levels of genes associated with chemoresistance and cell proliferation. The relative expression of genes involved with chemoresistance, such as APEX1, ERK1, MTDH, STAT3, and NFκB1, in addition to cell proliferation gene, i.e., MKI67 in (**A**) SF8628 (DIPG) and (**B**) CHLA-05-ATRT cells. The error bars represent the standard deviation in triplicate readings. The statistically significant data are represented with *, **, and *** for *p*-values less than 0.05, 0.01, and 0.001, respectively. UT: untreated; CDDP: cisplatin-treated; CDDP-As: cisplatin and iPSC-astrocyte-treated.

The increased expression of Caspase-9 (Figure 4D(ii)) and the decreased expression of Ki-67 (Figure 4B,D(i)) suggest that cisplatin exposure activated the apoptosis pathway in tumor cells. Although cisplatin-induced apoptosis and lowered tumor cell viability (Figure 2 and Figure 4), its cytotoxic efficacy was reduced in the presence of iPSC-astrocytes (Figure 3, Figure 5 and Figure 6), which may indicate the activation of potential cisplatin resistance pathways. To explore this further, cisplatin resistance markers, including NFκB1, APEX1, MTDH, ERK1, and STAT3, were investigated in tumor cells that survived cisplatin cytotoxicity (Figure 5, Figure 6 and Figure 7, Supplementary Figures S8–S10). The study conditions included untreated tumor cells (UT), cisplatin (CDDP)-treated cells, and tumor cells exposed to both cisplatin and astrocytes (CDDP-As) (Figure 4A).

In DIPG cells (Figure 5), STAT3 expression was low in untreated cells but markedly elevated in both CDDP and CDDP-As conditions. Similarly, NFκB1 and ERK1, which were negative in untreated cells, were strongly upregulated following exposure to CDDP or CDDP-As (Figure 5B), suggesting activation of their associated pathways. Both MTDH and APEX1 also showed increased expression per cell in CDDP and CDDP-As treated DIPG cells (Figure 5). For CHLA-05-ATRT cells, markers like APEX1, ERK1, MTDH, and NFκB1 were positive in the untreated condition, while STAT3 was negative (Figure 6).

In the CDDP and CDDP-As-exposed CHLA-05-ATRT conditions, the expression of these markers per cell decreased (Figure 6B). However, STAT3 expression per cell increased in both treated conditions. To further understand the alterations in the signaling of these essential markers associated with cisplatin resistance pathways, their mRNA level changes were investigated (Figure 7).

The experiment was performed by exposing SF8628 and CHLA-05-ATRT cells to a 25 μM concentration of cisplatin. In SF8628 (DIPG) cells exposed to CDDP and CDDP-As conditions, the expression of markers involved in cisplatin resistance and tumor promotion was upregulated (Figure 7A). In particular, the NFκB1 gene demonstrated a 4-fold increase in relative expression in CDDP-exposed cells. Interestingly, the expression of the MKI67 gene was upregulated in SF8628 cells exposed to CDDP-As, exceeding expression levels in both untreated and CDDP-exposed conditions. These findings strongly suggest that the interaction with astrocytes promotes chemoresistance in SF8628 cells (Figure 7A). In contrast, CHLA-05-ATRT cells exposed to CDDP and CDDP-As showed downregulation of markers involved in chemoresistance, such as APEX1, ERK1, MTDH, and STAT3 (Figure 7B). However, compared to the CDDP exposure condition, CDDP-As-exposed CHLA-05-ATRT cells showed upregulation of MTDH, NFκB1, MKI67, and ERK1 but downregulation of STAT3 and APEX1. Compared to the untreated condition, the NFκB1 gene was significantly upregulated in CDDP-As-exposed CHLA-05-ATRT cells but downregulated in the CDDP-exposed cells (Figure 7B).

Hence, SF8628 (DIPG) cells demonstrated notable cisplatin resistance in the presence of iPSC-astrocytes, as indicated by the increased SF8628 cell viability and the upregulation of resistance-associated markers (Figure 5 and Figure 7). Conversely, iPSC-astrocytes have an insignificant effect on the sensitivity of CHLA-05-ATRT cells towards cisplatin, as indicated by cell viability data (Figure 3 and Figure 4) and cisplatin resistance marker analysis (Figure 6 and Figure 7).

## 4. Discussion

DIPGs and ATRTs are rare and lethal pediatric brain tumors. Their infiltrative and heterogeneous nature limits the efficacy of currently employed therapeutic strategies [3,50], and their microenvironment can play a significant role in modulating drug response. Astrocytes are reported to either suppress or support tumor growth and chemoresistance in many brain tumors, including glioma and neuroblastoma [51,52,53,54,55]. Astrocytes inhibit the migration of minimally invasive gliomas while promoting the migration of highly invasive gliomas [56]. However, the influence of astrocytes on ATRTs and DIPGs remains unexplored. By examining the drug sensitivity of cancer cells in an astrocyte–tumor coculture system, we aimed to explore how astrocytes influence the tumor cells’ chemoresistance.

Here, we examined the effects of the crosstalk between malignant pediatric brain tumor cells and iPSC-astrocytes during cisplatin treatment on tumor cell proliferation and astrocyte activation. ATRT (CHLA-05-ATRT) and DIPG (SF8628) cells showed significantly higher proliferation upon coculture with iPSC-astrocytes, indicating a positive effect on tumor growth (Figure 1B). This interaction also upregulated STAT3 and GFAP in astrocytes (Figure 1C–E), suggesting the activation of astrocytes and their possible pro-tumorigenic function [27,57,58]. Cytokines such as IL-6 released by tumor cells may cause astrocyte activation [59,60]. These activated astrocytes release factors such as transforming growth factor beta (TGF-β) to support tumor growth [61]. Our data showed no significant change in brain-derived neurotrophic factor (BDNF) and C-C motif chemokine ligand (CCL5). BDNF is a growth factor that can alleviate tumor cell death and promote angiogenesis [62]. It suggests that iPSC-astrocytes promote tumor cell viability via pathways other than BDNF-related pathways. CCL5 is a member of the CC chemokine family that primarily acts as an inflammatory mediator and chemoattractant molecule in the central nervous system [63]. Unaltered CCL5 expression indicates that tumor cells did not activate astrocytes’ tumor-suppressive functions but instead promoted tumor-supportive properties, as evidenced by significant upregulation of STAT3 signaling following tumor cell exposure.

According to our drug dose–response data (Figure 2 and Supplementary Figure S6), ATRT and DIPG cells were more sensitive to cisplatin than methotrexate. However, drug sensitivity declined in tumor cells interacting with iPSC-astrocytes, particularly in DIPG cells (Figure 3 and Supplementary Figure S7). The presence of astrocytes surrounding the tumor cells reduced the cytotoxic efficacy of cisplatin. Therefore, we examined iPSC-astrocytes’ impact on some markers involved in potential cisplatin resistance pathways, including STAT3, NFκB1, ERK1/2, APEX1, and MTDH [20,64,65,66,67,68,69]. The STAT3 protein regulates tumor invasion and suppression of apoptosis [70], and the upregulation of this marker is linked to epithelial-to-mesenchymal transition [64]. STAT3 was weakly expressed in unexposed DIPG cells, but its expression was significantly elevated following exposure to CDDP and cisplatin–astrocyte (Figure 5B and Figure 7A). This result suggests a survival mechanism employed by the tumor cells to adapt and counteract the effects of drug treatment. STAT3 activation is associated with acquired drug resistance in cancer cells [71]. The downstream targets of STAT3 include anti-apoptotic genes (BCL-2, BCL-XL, MCL-1) [72], cell survival pathway genes (Cyclin-D1, Survivin) [73,74], drug efflux (MDR1) [75,76], and maintenance genes of cancer stem cells (CD133) [77,78]. Therefore, STAT3 activation in these cancer cells might help them prevent apoptosis and enhance self-renewal. Similarly, NFκB1 can stimulate the expression of anti-apoptotic genes and promote cell proliferation, thereby protecting DNA-damaged cells from apoptosis [79]. In addition, NFκB1 drives the expression of drug efflux pumps, lowering intracellular drug concentrations [80,81]. NFκB1 also diminishes reactive oxygen species by inducing manganese superoxide dismutase [82].

STAT3 interacts with NFκB1 at multiple levels; it extends NFκB1–nuclear retention [83], and together, they control the ability of cancer cells to resist apoptosis [84]. Like STAT3 expression, the NFκB1 marker was almost negative in untreated DIPG and ATRT cells; however, in the exposed conditions, NFκB1 was positive and markedly upregulated (Figure 5, Figure 6 and Figure 7). When CHLA-05-ATRT cells were exposed to cisplatin during their coculture with astrocytes, NFκB1 expression increased 2.6-fold compared to monoculture cisplatin conditions (Figure 7). Evidence suggests that tumor cells release cytokines (e.g., IL-6) to activate astrocytes via JAK/STAT and GFAP signaling [59]. In turn, activated astrocytes secrete factors such as TGF-β, which amplify NFκB1 signaling in tumor cells through a feedforward mechanism, promoting their chemoresistance [25,61,85,86,87]. Our findings demonstrated significant upregulation of NFκB1 under CDDP and CDDP-As conditions. Hence, targeting NFκB1 and STAT3 signaling may improve the cisplatin sensitivity of ATRTs and DIPGs. However, further comprehensive studies are required to validate these findings and elucidate the underlying mechanisms.

iPSC-astrocytes’ role in mitigating cancer cell death was further evidenced by the upregulation of extracellular signal-regulated kinase 1 (ERK1) and the proliferation marker Ki-67 (MKI67). ERK1 supports tumor cell survival and invasiveness via the RAF-MEK-ERK1 pathway, which is upregulated in drug-resistant tumor cells [88,89,90]. High MKI67 expression is strongly correlated with aggressive tumor behavior, rapid growth, and poor prognosis [91,92,93]. The robust expression of ERK1 and MKI67 signals in ATRT and DIPG cells underscores their aggressive nature. Notably, the expression of ERK1 in DIPG was further elevated in the presence of iPSC-astrocytes, implying that interactions with iPSC-astrocytes enhance drug resistance and promote tumor survival in DIPG (Figure 5 and Figure 7A). However, in CHLA-05-ATRT cells exposed to CDDP and CDDP-As conditions, the expression of both ERK1 and MKI67 decreased, implying their heightened sensitivity to cisplatin (Figure 6 and Figure 7B).

**Figure 8:**
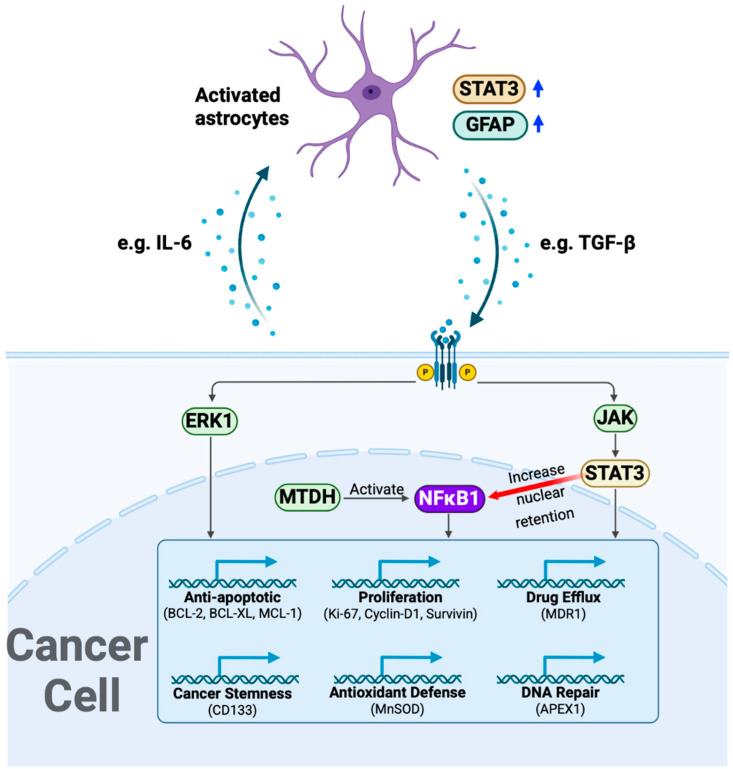
A schematic representation of cisplatin resistance mechanisms in pediatric brain tumor cells mediated by astrocytes. Tumor-derived chemokines (e.g., IL-6) activate brain-resident astrocytes, as indicated by the upregulation of STAT3 and GFAP. These primed astrocytes, in turn, promote tumor progression by secreting pro-tumorigenic factors such as TGF-β. Subsequent activation of ERK1, STAT3, and NFκB1 in tumor cells enhances cisplatin resistance through multiple mechanisms, including increased anti-apoptotic signaling, proliferation, drug efflux, cancer stemness, antioxidant defense, and DNA repair. Additionally, STAT3 facilitates NFκB1 nuclear retention, while MTDH, a positive regulator of NFκB1 and MEK/ERK signaling, further amplifies NFκB1 activation, collectively driving drug resistance. IL-6: interleukin-6, STAT3: signal transducer and activator of transcription 3, GFAP: Glial fibrillary acidic protein, TGF-β: transforming growth factor beta, NFκB1: Nuclear factor-κB 1, MTDH: Metadherin, ERK1: Extracellular signal-regulated kinase 1.

We also examined a potential DNA repair mechanism activated by the alkylating agent cisplatin. Apurinic/apyrimidinic endonuclease-1 (APEX1) is an enzyme involved in the base excision repair pathway that can get activated upon exposure to chemotherapy [94]. APEX1 expression was highly upregulated in SF8628 cells under CDDP and CDDP-As conditions (Figure 5 and Figure 7A) while downregulated in CHLA-05-ATRT cells under the same conditions (Figure 6 and Figure 7B). Metadherin (MTDH) also exhibited an expression pattern like that of APEX1 in the treated DIPG cells. MTDH promotes tumor proliferation by inhibiting transcriptional factor FOXO1 [95] and activating MEK/ERK and NFκB1 pathways [96], thereby driving tumor progression. The elevated expression of APEX1 and MTDH in DIPG cells highlights their cisplatin-resistant properties, suggesting that targeting APEX1 and MTDH could improve cisplatin cytotoxic efficacy [66,94,97]. Figure 8 provides a schematic illustration of the cisplatin resistance mechanisms developed by pediatric brain tumor cells under the influence of astrocytes.

While the coculture model of iPSC-astrocytes and tumor cells is valuable for distinguishing astrocytes’ contribution to drug resistance independent of other factors of the tumor microenvironment, it has limitations in replicating the complexity of in vivo conditions. Complementary in vivo experiments, including xenograft and humanized mouse models, can be employed to address these gaps. Integrating coculture findings with in vivo models can ensure a comprehensive approach with physiological relevance for better translational outcomes. Future experiments may also utilize CRISPR-based strategies to knockout or knockdown key resistance genes in tumor cells or immune-modulatory genes in iPSC-astrocytes to assess how these genes influence the drug sensitivity of cocultured ATRT or DIPG cells. Disrupting astrocyte-tumor crosstalk by targeting IL-6, TGF-β, and their receptors with monoclonal antibodies may suppress astrocyte activation and help delay the development of drug resistance [98,99].

In summary, this study explored the impact of iPSC-astrocytes on the cisplatin sensitivity of CHLA-05-ATRT and SF8628 (DIPG) cells. While iPSC-astrocyte coculture increased the viability of both cell lines following cisplatin treatment, gene expression analysis revealed distinct patterns between them. These findings suggest that CHLA-05-ATRT cells are more sensitive to cisplatin than SF8628 cells and that the rescue effect of iPSC-astrocytes was not significant for CHLA-05-ATRT. One possible explanation for this difference lies in the distinct mutational landscapes of the tumors. Aberrant transcriptional programs of ATRT cells, caused by the inactivation of the chromatin remodeling function of SMARCB1, potentially undermine DNA repair pathways required to correct cisplatin-induced DNA lesions [100]. On the other hand, DIPGs consistently exhibit a functional DNA repair repertoire to withstand such damages [101,102]. However, beyond intrinsic differences in chemosensitivity, our findings highlight two potential strategies for improving chemotherapy efficacy or delaying the development of acquired drug resistance: first, by eliminating the pro-tumor function of astrocytes, and second, by disrupting the communication between astrocytes and cancer cells. Nonetheless, further research—both to identify key targets and to validate them in vivo—is essential to provide robust evidence for guiding therapeutic strategies.

## 5. Conclusions

This study investigated the effects of iPSC-astrocytes on the anti-tumor activity of cisplatin and delved into the potential cisplatin resistance mechanisms in CHLA-05-ATRT and SF8628 (DIPG) cells. The interaction of these tumor cells with iPSC-astrocytes upregulated the tumor-promoting state of astrocytes. The tumor cells in crosstalk with iPSC-astrocytes displayed reduced sensitivity to cisplatin. Moreover, this interaction upregulated the expression of potential markers involved in cisplatin resistance pathways. Interestingly, markers such as ERK1, STAT3, and NFκB1, with negligible expression in SF8628 cells, became significantly upregulated upon exposure to cisplatin and astrocytes. Contrarily, in CHLA-05-ATRT cells, these chemoresistance markers were downregulated except NFκB1 when exposed to cisplatin and astrocytes. The expression and cell viability data indicate that CHLA-05-ATRT cells were more sensitive to cisplatin than SF8628 cells. These findings underscore the need to modulate astrocyte–tumor cell interactions to enhance treatment efficacy.

## Data Availability

The datasets generated and analyzed during the current study are in this paper’s main text and Supplementary Materials. The corresponding authors will provide additional relevant data upon reasonable request.

## Supplementary Materials

The following supporting information can be downloaded at https://www.mdpi.com/article/10.3390/cancers17060997/s1, Figure S1: Differentiation of human iPSC-astrocytes; Figure S2: Characterization of iPSC-astrocytes; Figure S3: Expression of astrocyte-associated markers; Figure S4: The percent expression of STAT3 in iPSC-astrocytes exposed to cancer cells; Figure S5: The gating population of Figure 1D; Figure S6: Relative viability study in ATRT and DIPG cells exposed to the methotrexate at various concentrations; Figure S7: Cytotoxicity of anti-cancer drugs affected by astrocyte–tumor cell crosstalk; Figure S8: Effects of cisplatin exposure in the presence of iPSC-astrocytes on the expression of NFκB1 and APEX1 markers; Figure S9: Effects of cisplatin exposure in the presence of iPSC-astrocytes on the expression of ERK1 and MTDH; Figure S10: The expression of the STAT3 marker; Table S1: List of antibodies; Table S2: Sequences of forward and reverse primers for RT-PCR; Supplementary Table S3: A summary table of astrocyte characterization markers. Refs [103,104,105,106,107] are cited in the Supplementary Table S3.
