# Supplementary material for: Effects of Induced Pluripotent Stem Cell-Derived Astrocytes on Cisplatin Sensitivity in Pediatric Brain Cancer Cells
Source: Cancers (Basel). 2025 Mar 16;17(6):997. doi: 10.3390/cancers17060997 (PMC11940393; doi:10.3390/cancers17060997)
Supplement: Supplementary file 1 [file cancers-17-00997-s001.zip › cancers-3474212-supplementary.pdf]

Supplementary Information

Effects of Induced Pluripotent Stem Cell-Derived Astrocytes on Cisplatin Sensitivity in Pediatric Brain Cancer Cells

Sonia Kiran[1], Yu Xue[1], Drishty B. Sarker[1], and Qing-Xiang Amy Sang[1,2,*]

[1]Department of Chemistry and Biochemistry, Florida State University, Tallahassee, FL 32306-4390, USA.

[2]Institute of Molecular Biophysics, Florida State University, Tallahassee, FL 32306-4380, USA.

[*]Correspondence: qxsang@chem.fsu.edu ; Tel.: +1-850-644-8683; Fax: +1-850-644-8281

Contact information for other authors:

Sonia Kiran: skiran@fsu.edu

Yu Xue: yx21@fsu.edu

Drishty B. Sarker: ds22@fsu.edu

Supplementary Figure S1. Astrocyte differentiation from induced pluripotent stem cells (iPSCs). (A) iPSC-derived astrocytes **(i)** Phase-contrast images: The morphology of cells on days 6, 13, and 40 of differentiation into astrocytes. The scale bar of the image taken on day 6 and day 13 is 40 µm, and on day 40 is 100 µm**. (ii)** Immunocytochemistry images display the expression of astrocyte-associated markers GFAP, S100B, and CSPG with a scale bar of 100 µm. **(B)** Flow cytometry quantifies the astrocyte-specific markers such as GFAP, S100B, and CSPG in iPSC-Astrocytes. (**C**) Normal Human astrocytes (NHA) (**i**) Phase-contrast images: The morphology of NHA on days 2, 4, and 7 of cell culture. The scale bar is 100 µm. **(ii)** Immunocytochemistry images show the expression of astrocyte-associated markers GFAP, S100B, and CSPG with a scale bar of 100 µm. GFAP: glial fibrillary acidic protein, S100B: S100 calcium-binding protein B, and CSPG: chondroitin sulfate proteoglycan. Anti-mouse and anti-rabbit IgGs are the isotype control cells treated with secondary antibodies only to exclude the non-specific binding.

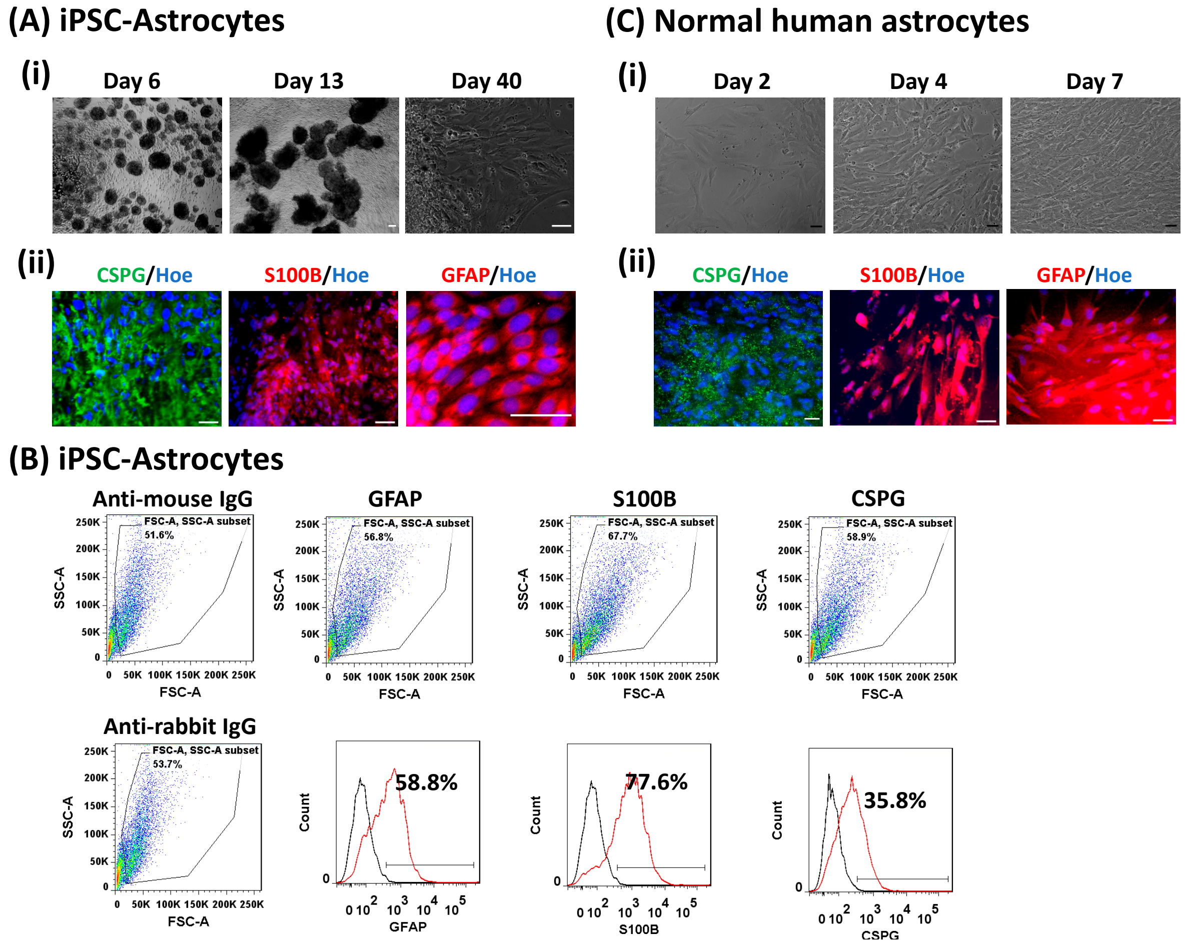

Supplementary Figure S2. Characterization of iPSC-astrocytes. (A) Phase-contrast images during the differentiation of iPSCs into astrocytes. Scale bar: 40 μm for days 3, 5, 7, and day 14 and 100 μm for day 25 and day 40. **(B)** Expression of astrocyte-associated markers such as **GFAP, S100B, CSPG, Vim,** and **HA in** iPSC-astrocytes**.** scale bar: 100 μm. **GFAP**: glial fibrillary acidic protein; **CSPG**: chondroitin sulfate proteoglycan; **HA**: hyaluronic acid; **Vim**: vimentin; **S100B**: S100 calcium-binding protein B)**.**

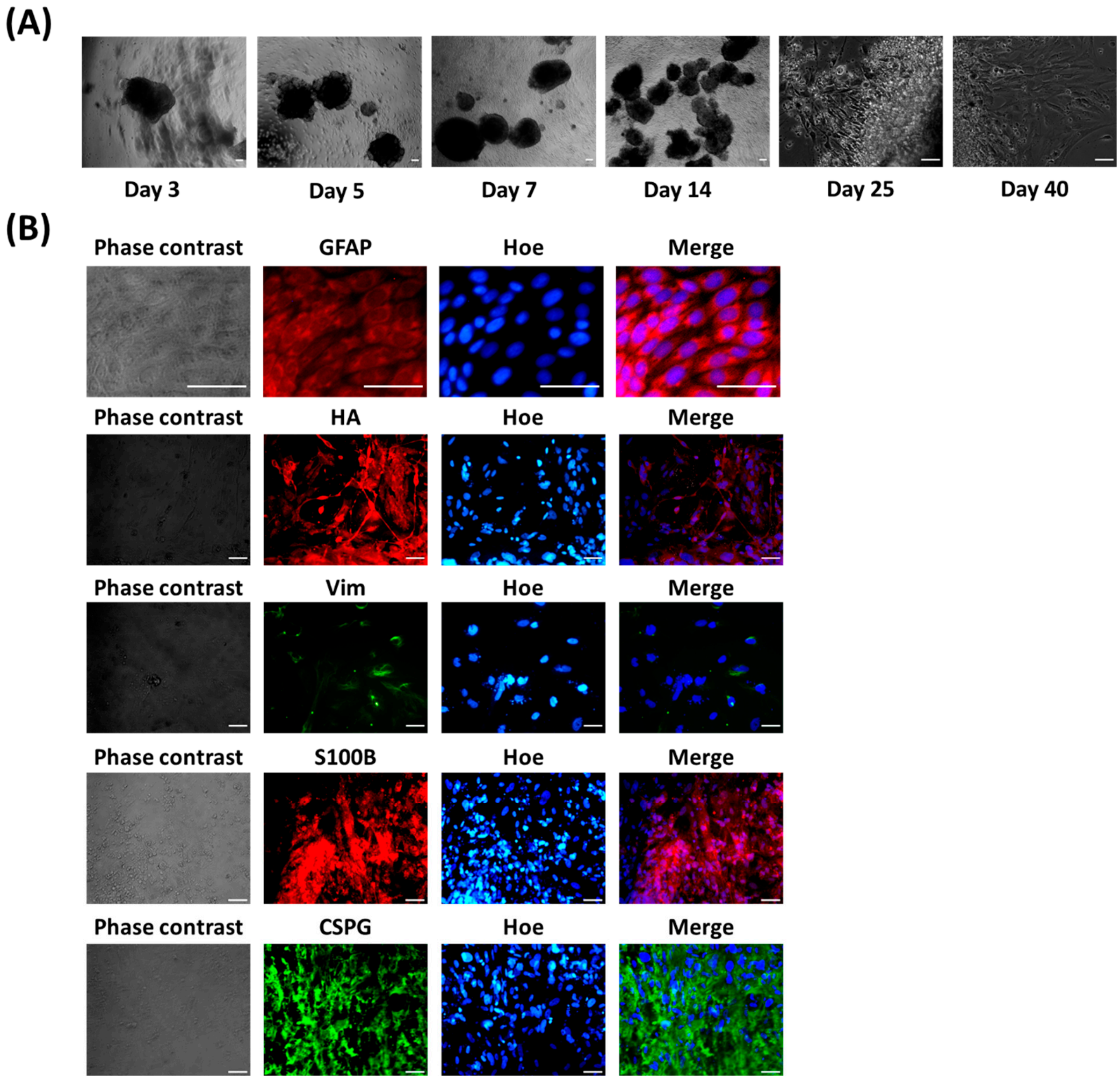

Supplementary Figure S3. Expression of astrocyte-associated markers such as **GFAP, S100B, CSPG, Vim,** and **HA in** Normal human astrocytes. scale bar: 100 μm. **GFAP**: glial fibrillary acidic protein; **CSPG**: chondroitin sulfate proteoglycan; **HA**: hyaluronic acid; **Vim**: vimentin; **S100B**: S100 calcium-binding protein B**.**

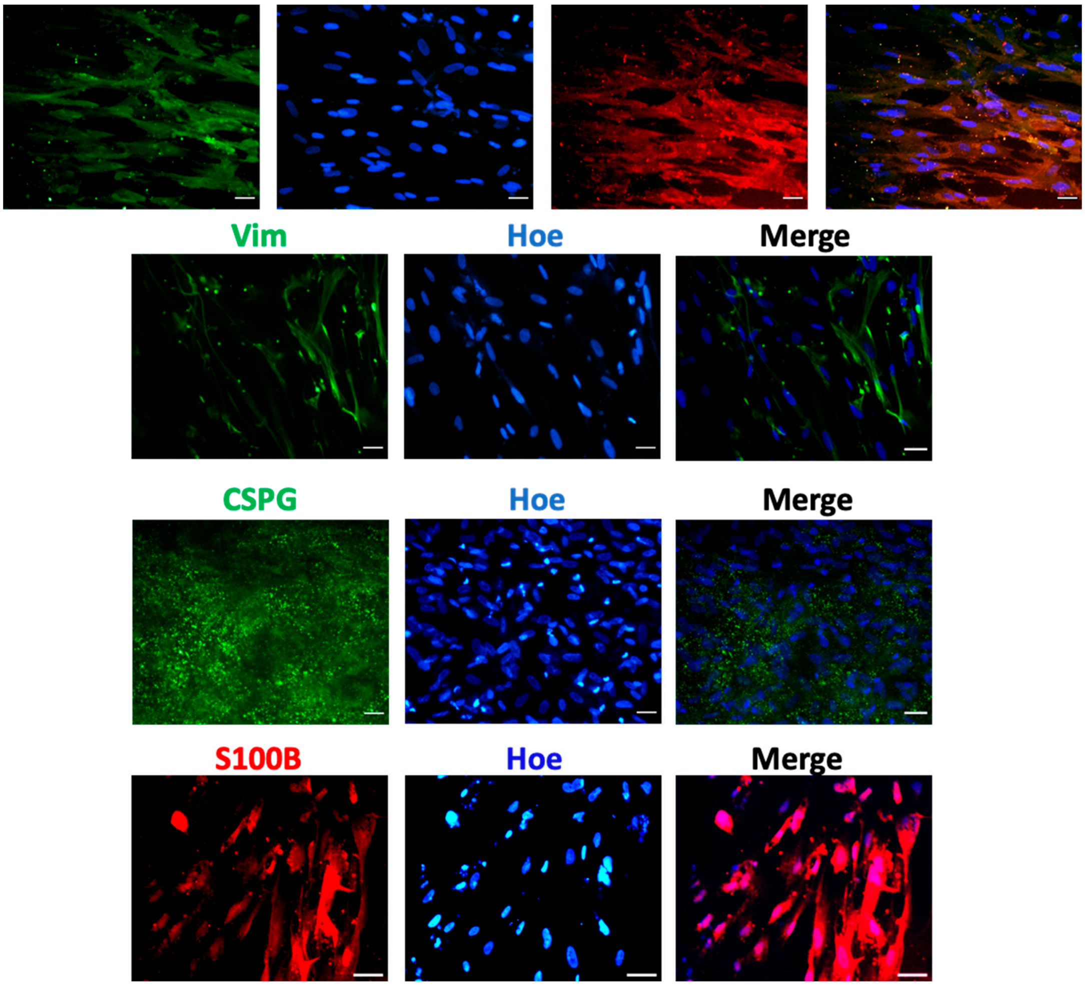

Supplementary Figure S4: **(A)** The percent expression of STAT3 in iPSC-Astrocytes exposed to cancer cells **(B)** The quadrants showing marker expression in various control conditions for **Figure 1-D(ii). (C)** The gating population of NHA expressing STAT3 and GFAP.

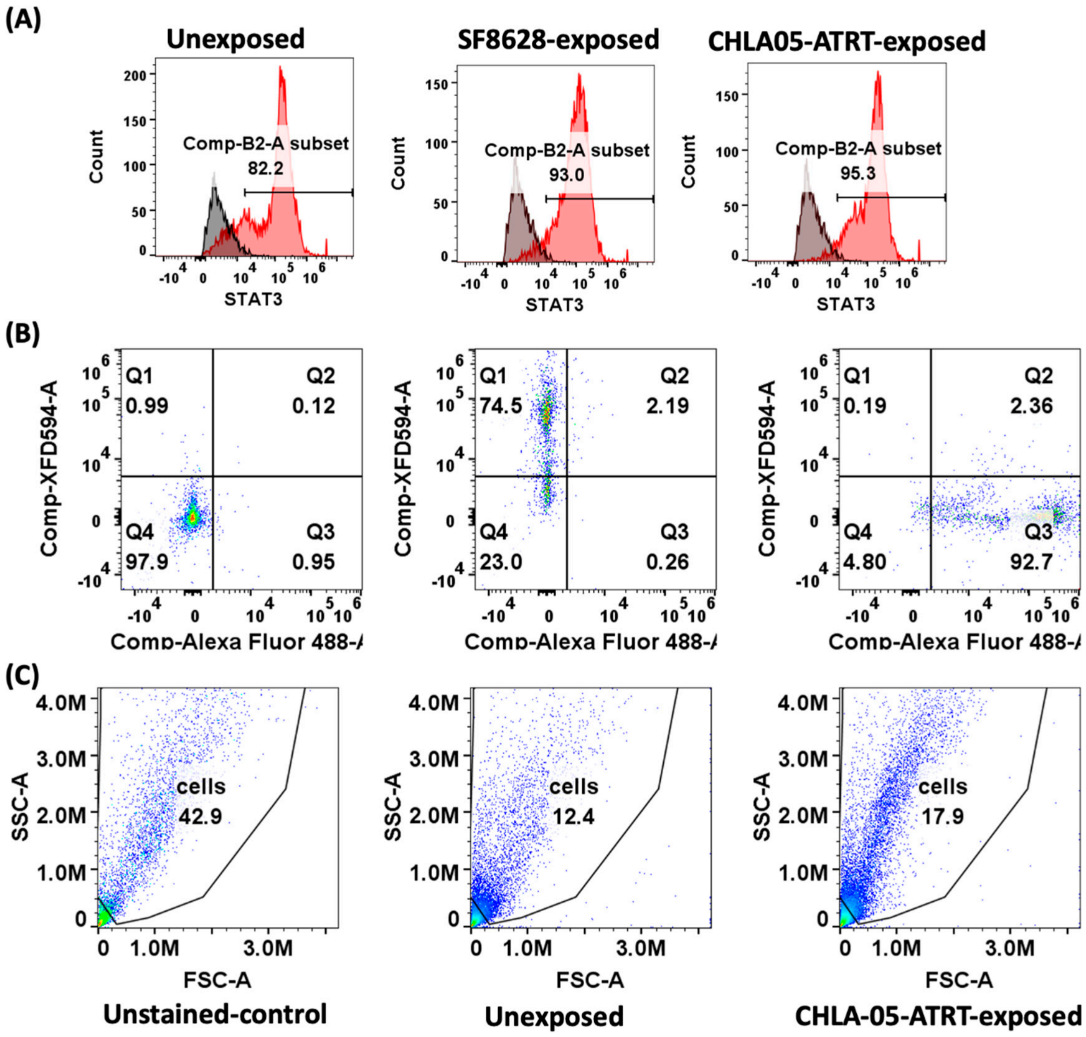

Supplementary Figure S5: **(A)** The gating population of **Figure 1-D-(i)** **(B)** The gating population of **Figure 1-D-(ii).**

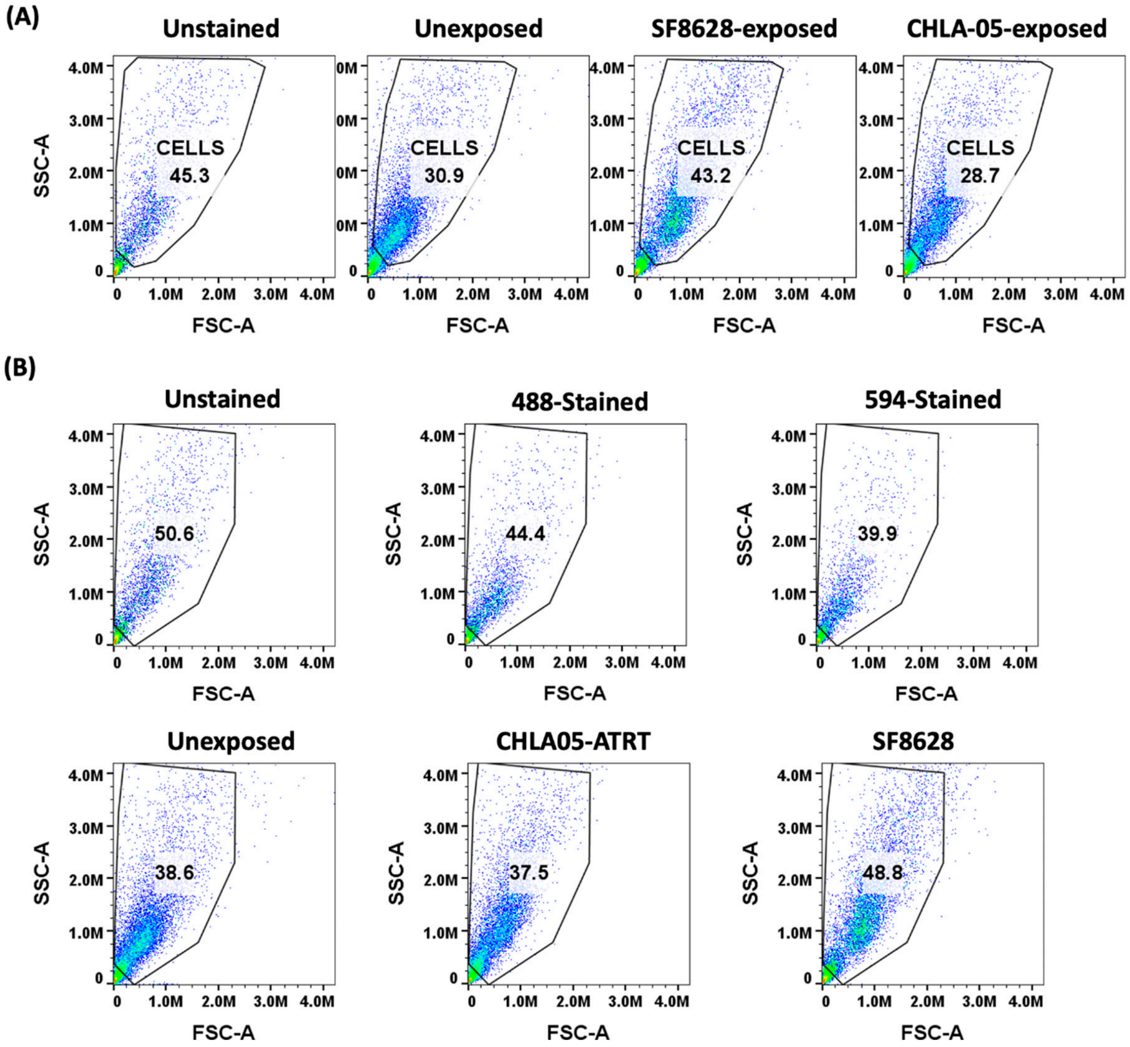

Supplementary Figure S6: Relative viability study in ATRT and DIPG cells exposed to anti-cancer drugs at various concentrations. (A) Bar graphs indicate the percent cytotoxic effects of **Methotrexate (MTX)** on the viability of SF8628 and CHLA-05-ATRT cells. **(B)** The dose-response curve of MTX indicates its cytotoxicity towards **(i)** SF8628 and **(ii)** CHLA-05-ATRT cells. **(C)** The IC_{10}, IC_{50}, and IC_{90} of Methotrexate and cisplatin towards CHLA-05-ATRT and SF8628 cells. The error bars represent triplicate readings, and statistical significance is shown by *, **, and *** for p-values less than 0.05, 0.01, and 0.001, respectively. The inhibition dose-response curves are plotted using Biorender.com.

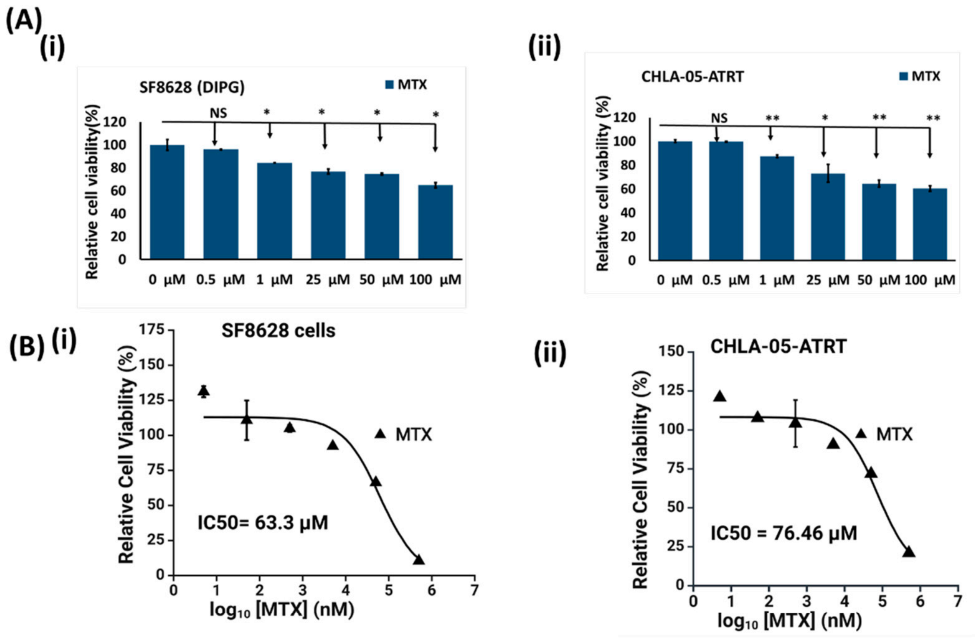

(C) (i)

SF8628	IC10 (μM)	IC50 (μM)	IC90 (μM)
CDDP	0.31	2.78	25.02
MTX	7.03	63.3	569.7

(ii)

CHLA-05-ATRT	IC10 (μM)	IC50 (μM)	IC90 (μM)
CDDP	0.41	3.7	33.3
MTX	8.49	76.46	688.14

Supplementary Figure S7: Cytotoxicity of anti-cancer drugs affected by astrocyte-tumor cell crosstalk. (**A**) The diagram represents the coculture system of iPSC-astrocytes and tumor cells. The effect of astrocytes on the cisplatin and methotrexate anti-tumor activity in **(B)** SF8628 and (**C**) CHLA-05-ATRT cells. **(B)** Upon exposure to MTX at 25 μM and 100 μM, the viability of **(i)** DIPG cells and **(ii)** CHLA05-ATRT cells increased

significantly in the coculture with astrocytes. **(C)** The effects of both CDDP and MTX at 25 µM and 100 µM on the viability of **(i)** SF8628 and **(ii)** CHLA-05-ATRT cells. The error bars represent the standard deviation in triplicate readings, and statistical significance is shown by *, **, and *** for p-values less than 0.05, 0.01 and 0.001 respectively.

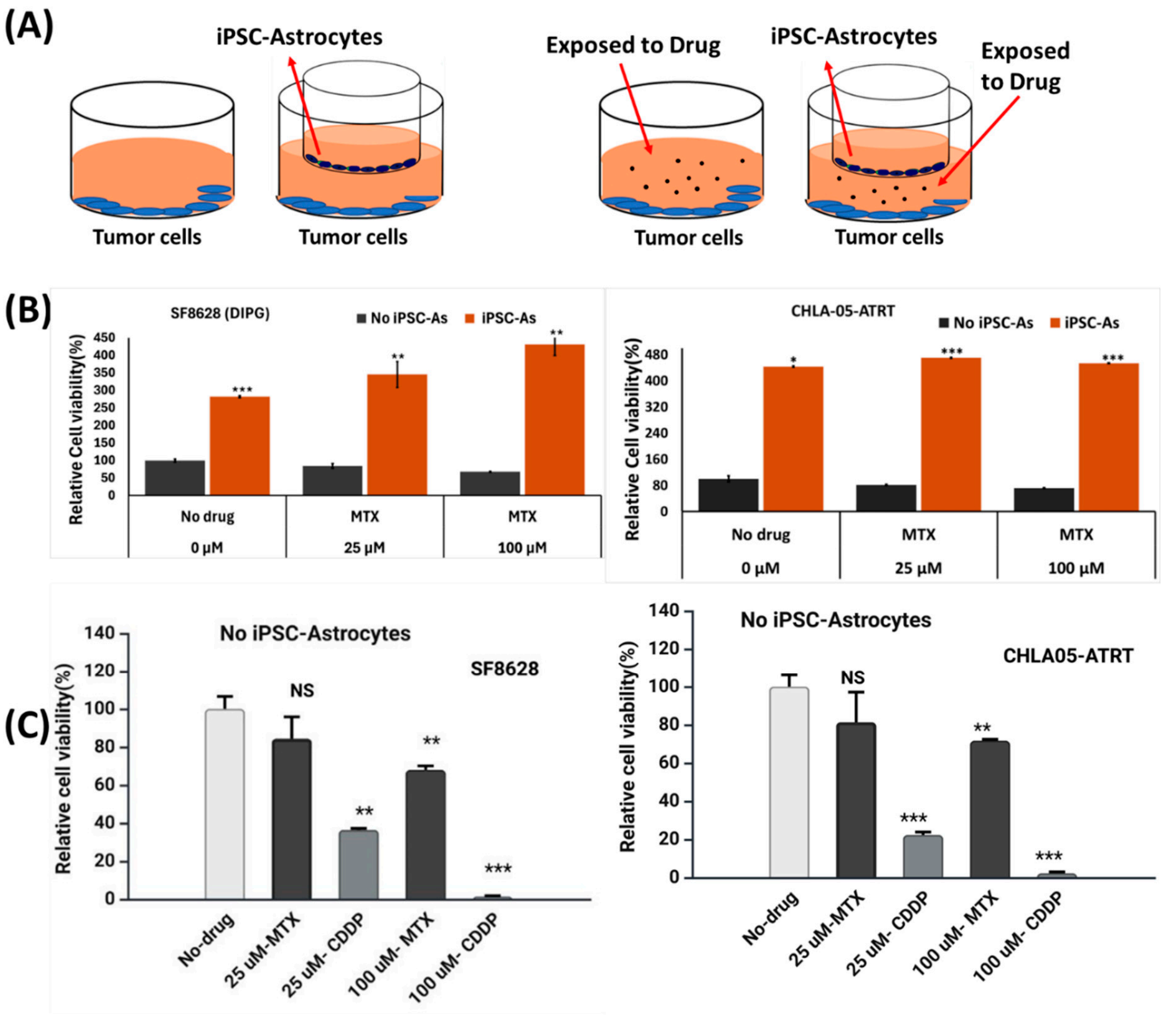

Supplementary Figure S8: Effects of cisplatin exposure in the presence of iPSC-astrocytes on the expression of NFκB1 and APEX1 markers. The expression of the NFκB1 and APEX1 markers in **(A)** CHLA-05-ATRT and **(B)** SF8628 cells in untreated, cisplatin, and cisplatin-astrocytes exposed pediatric brain tumor cells. The images were captured with 200x magnification, and the scale bar is 100 µm. APEX1: Apurinic/apyrimidinic endonuclease 1, NFκB1: Nuclear factor-κB1.

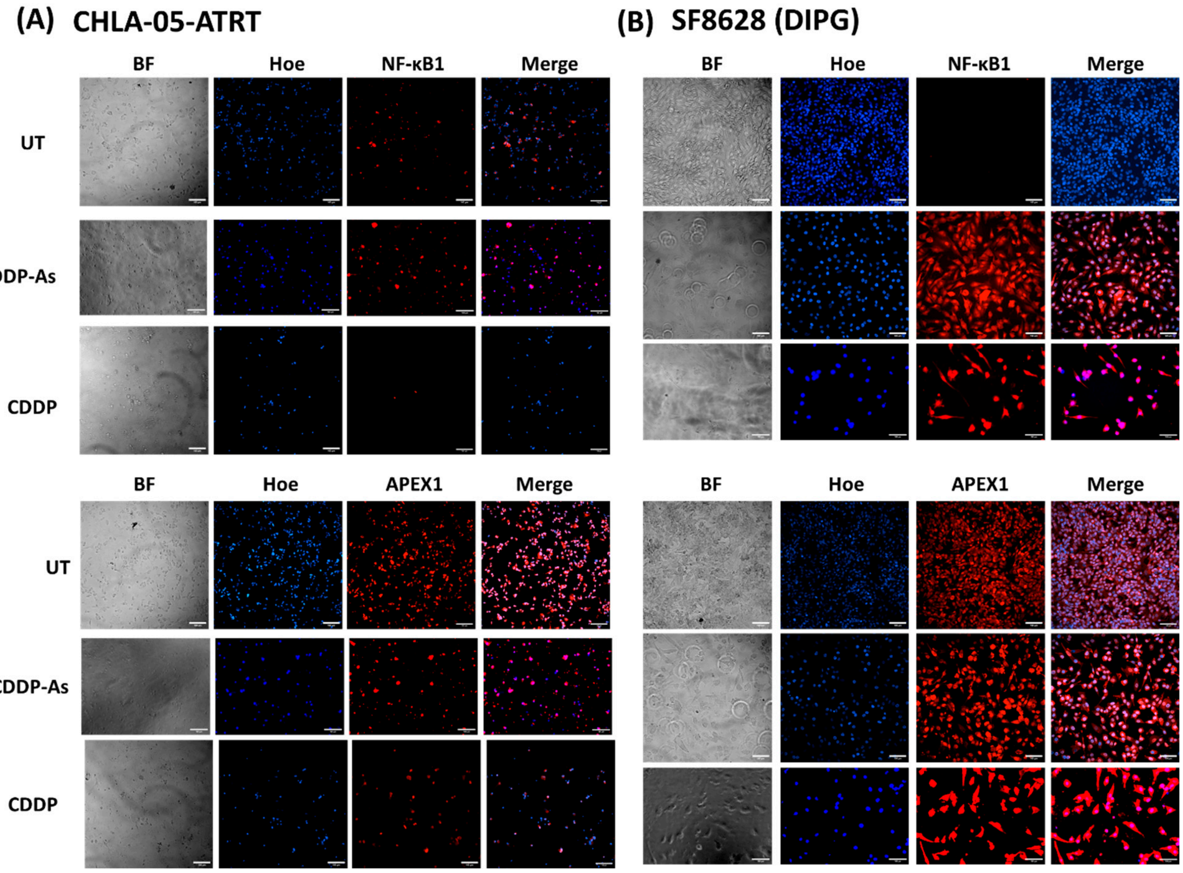

(A) CHLA-05-ATRT
(B) SF8628 (DIPG)
BF
Hoe
NF-κB1
Merge
UT
CDDP-As
CDDP
BF
Hoe
APEX1
Merge
UT
CDDP-As
CDDP

Supplementary Figure S9: Effects of cisplatin exposure in the presence of iPSC-astrocytes on the expression of the **(A)** ERK1 and **(B)** MTDH markers in **(i**) CHLA-05-ATRT and **(ii)** SF8628 cells under three conditions, i.e., untreated, cisplatin and cisplatin-astrocytes exposed pediatric brain tumor cells. The images were captured with 200x magnification, and the scale bar is 100 µm. ERK1: Extracellular signal-regulated kinase 1, MTDH: Metadherin. Hoe: Hoechst 33342.

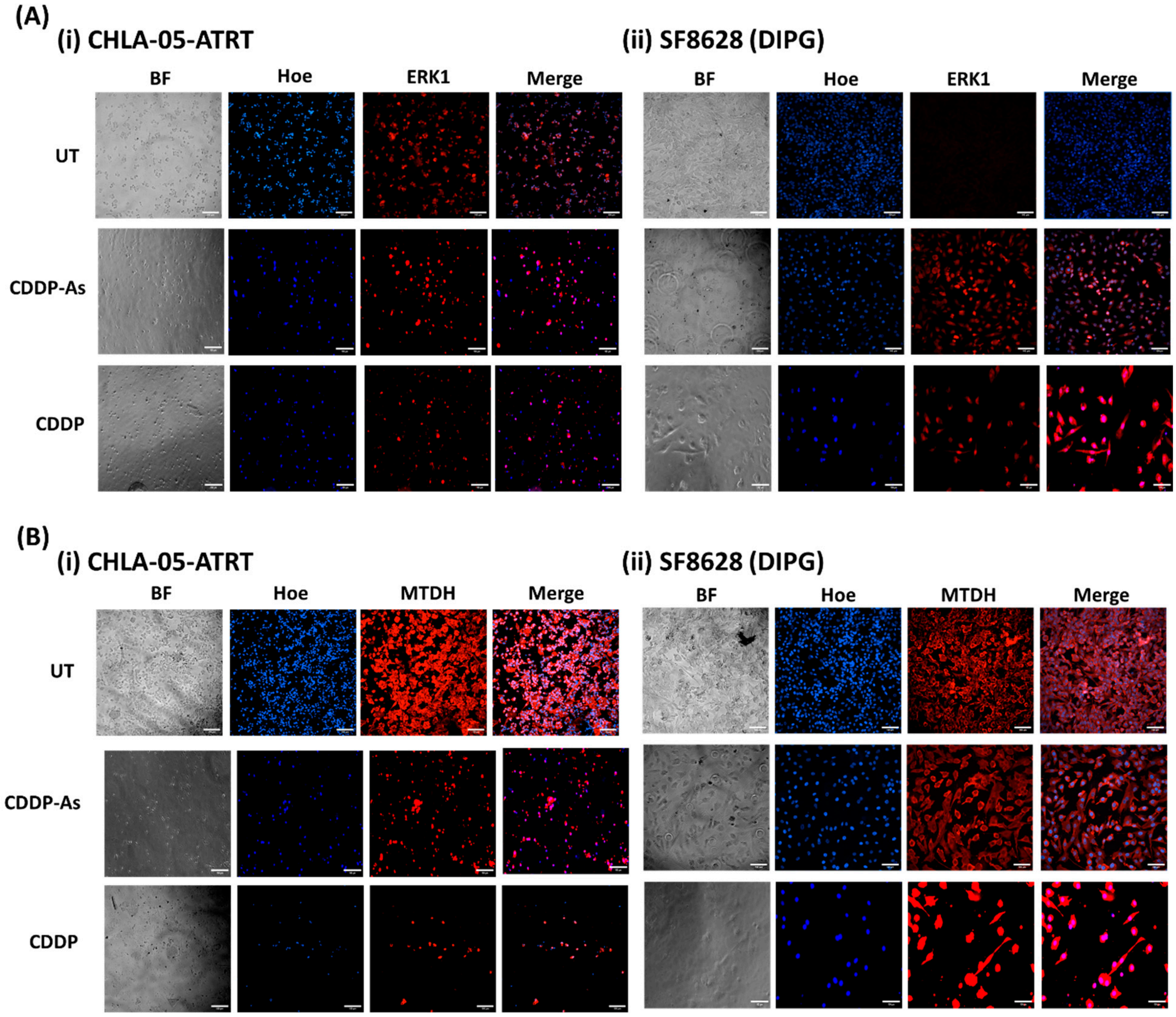

Supplementary Figure S10. The expression of the STAT3 marker in (A) CHLA-05-ATRT and **(B)** SF8628 cells under the three conditions, i.e., untreated, cisplatin, and cisplatin-astrocytes, exposed pediatric brain tumor cells. The images were captured with 200x magnification, and the scale bar is 100 μm. Hoe: Hoechst 33342, STAT3: Signal transducer and activator of transcription 3.

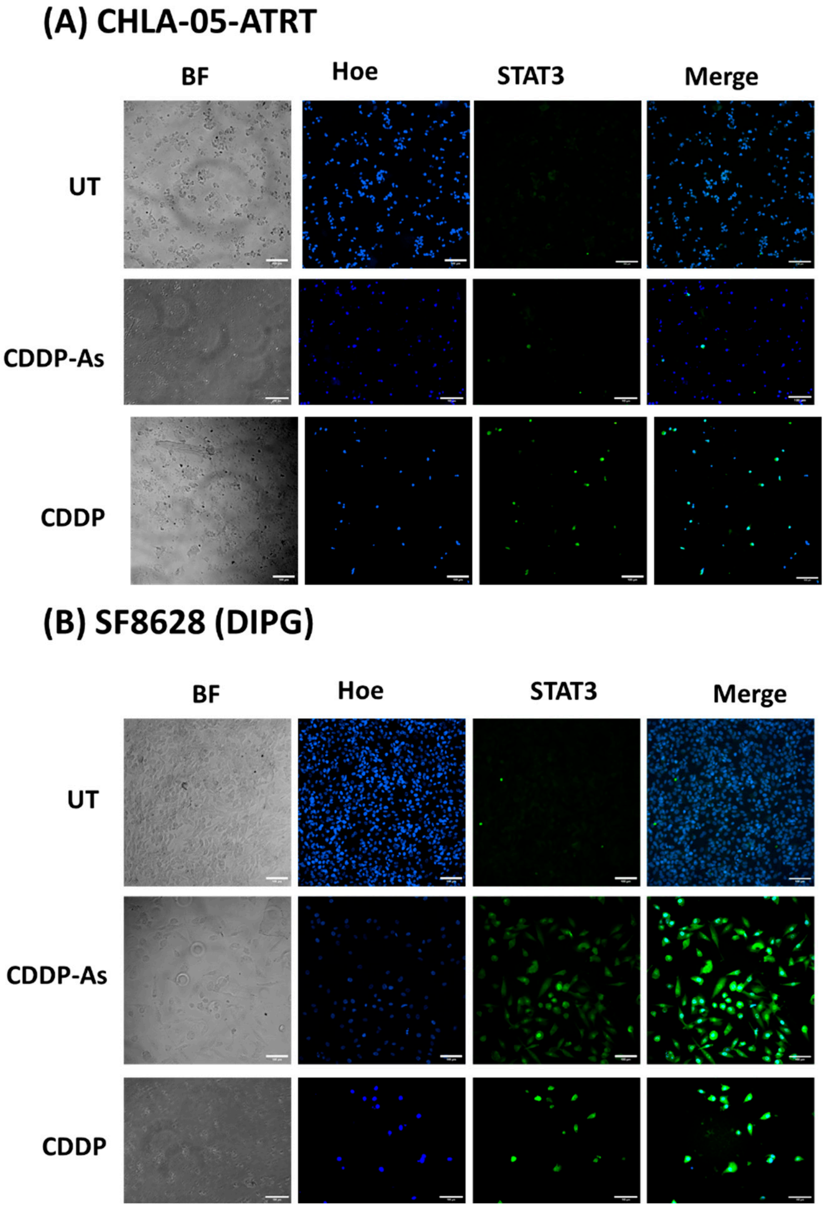

Supplementary Table S1. List of antibodies.

Antibodies	Origin/Isotype	Supplier/Cat #	Dilution for ICC and flow cytometry
**NFKB1**	Rabbit pAb	ABclonal, A11160	1:150
**APEX1**	Rabbit pAb	ABclonal, A2587	1:150
**ERK1/2**	Rabbit mAb	ABclonal, A4782	1:150
**MTDH**	Rabbit pAb	ABclonal, A5887	1:100
**Caspase9**	Rabbit pAb	Sino biological, 102185-T38	1:300
**Ki67**	Mouse mAb	Sino biological, 100130-MM22	1:200
**STAT3**	Mouse IgG	DSHB, 2f12-s	2.5 ug/mL
**S100B**	Mouse IgG	Sino biological, 100508-MM03	1:500
**GFAP**	Rabbit pAb	Sino biological, 206278-T44	1:1000
**Vimentin**	Mouse monoclonal IgM	Santa Cruz Biotechnology Sc- 80975	1:100
**Hyaluronic acid**	Sheep IgG	Invitrogen, PA1-85561	5ug/mL
**Alexa Fluor™ 488**	Goat Anti-Mouse IgG, IgM, IgA (H+L)	Invitrogen, A-10667	1:200
**Alexa Fluor™ 594**	Goat Anti-rabbit IgG (H+L)	Invitrogen, A-11012	1:200
**Alexa Fluor™ 568**	Goat anti-Mouse IgG (H+L)	Invitrogen, A-11004	1:200

Supplementary Table S2. Sequences of forward and reverse primers for RT-PCR.

Gene	Orientation	Sequences (5' to 3')
**APEX1**	F	AGGAGCTGCCTGGACTCTCTC
	R	CTCATCGCCTATGCCGTAAGA
**STAT3**	F	CTCTCCTGTGCGTATGGGAAC
	R	CTGAGGCAAGGTGGTTTTGAG
**ERK1**	F	AAGTTGCTGAAAAGCCAGCAG
	R	GGGCTTTAGATCTCGGTGGAG
**MTDH**	F	GGTTGTCATGGGCCAAATTAAC
	R	AAAATGCTTTGGTGCAGGAGA
**NFκB1**	F	TGAGTCCTGCTCCTTCCAAAA
	R	TCGGTGTAGCCCATTTGTCTC
**MKI67**	F	ATACCCACCCATTTCCACCAT
	R	CCTGGGAGGCGAAAAAGTAAA
**GFAP**	F	AGGACCTGCTCAATGTCAAGC
	R	CAGGTTGGAGAAGGTCTGCAC
**BDNF**	F	AATTTTGCTCCCCAGTGAAGG
	R	GGCTCCCAACTTGACTTCTCC
**CCL5**	F	AATTTGCCTGTTTCTGCTTGC
	R	GTGGTAGAATCTGGGCCCTTC

Supplementary Table S3. A summary table of astrocyte characterization markers.

Marker	*Full Name*	*Association with Astrocytes*	*Reference*
GFAP	Glial Fibrillary Acidic Protein	GFAP is an intermediate filament protein predominantly expressed in astrocytes. It plays a crucial role in maintaining astrocyte mechanical strength and shape. GFAP expression is commonly used to identify astrocytes in both healthy and diseased states.	[103]
CSPG	Chondroitin Sulfate Proteoglycan	CSPGs are components of the extracellular matrix produced by various cell types, including astrocytes. They are involved in modulating cell adhesion, migration, and neurite outgrowth. In the context of CNS injury, reactive astrocytes secrete CSPGs, contributing to the formation of the glial scar, which can inhibit axonal regeneration.	[104]
HA	Hyaluronic Acid	HA is a glycosaminoglycan found in the extracellular matrix. While not exclusive to astrocytes, they synthesize and secrete HA, contributing to the extracellular environment's structural integrity. HA plays roles in cell proliferation, migration, and tissue hydration. Its accumulation in the glial scar after injury can influence neural repair processes.	[105]
Vim	Vimentin	Vimentin is an intermediate filament protein expressed in various cell types, including astrocytes. During development and in response to injury, astrocytes upregulate vimentin expression. It is often co-expressed with GFAP in reactive astrocytes, indicating cytoskeletal reorganization during astrogliosis.	[106]
S100B	S100 Calcium-Binding Protein B	S100B is a calcium-binding protein predominantly expressed by astrocytes. It is involved in the regulation of protein phosphorylation, cell proliferation, and differentiation. Elevated levels of S100B in biological fluids are considered markers of astrocytic activation or injury and have been studied as biomarkers in various neurological conditions.	[107]
